# Single-Cell Analysis of Multiple Steps of Dynamic NF-κB Regulation in Interleukin-1α-Triggered Tumor Cells Using Proximity Ligation Assays

**DOI:** 10.3390/cancers11081199

**Published:** 2019-08-16

**Authors:** Christin Mayr-Buro, Eva Schlereth, Knut Beuerlein, Ulas Tenekeci, Johanna Meier-Soelch, M. Lienhard Schmitz, Michael Kracht

**Affiliations:** 1 Rudolf-Buchheim-Institute of Pharmacology, Justus Liebig University, D-35392 Giessen, Germany; 2 Institute of Biochemistry, Justus Liebig University, D-35392 Giessen, Germany

**Keywords:** tumor heterogeneity, NF-κB, interleukin-1 signaling, transcription, posttranscriptional gene regulation, P-bodies, DCP1a, proximity ligation assays, single cell analysis

## Abstract

The frequently occurring heterogeneity of cancer cells and their functional interaction with immune cells in the tumor microenvironment raises the need to study signaling pathways at the single cell level with high precision, sensitivity, and spatial resolution. As aberrant NF-κB activity has been implicated in almost all steps of cancer development, we analyzed the dynamic regulation and activation status of the canonical NF-κB pathway in control and IL-1α-stimulated individual cells using proximity ligation assays (PLAs). These systematic experiments allowed the visualization of the dynamic dissociation and re-formation of endogenous p65/IκBα complexes and the nuclear translocation of NF-κB p50/p65 dimers. PLA combined with immunostaining for p65 or with *NFKBIA* single molecule mRNA-FISH facilitated the analysis of (i) further levels of the NF-κB pathway, (i) its functionality for downstream gene expression, and (iii) the heterogeneity of the NF-κB response in individual cells. PLA also revealed the interaction between NF-κB p65 and the P-body component DCP1a, a new p65 interactor that contributes to efficient p65 NF-κB nuclear translocation. In summary, these data show that PLA technology faithfully mirrored all aspects of dynamic NF-κB regulation, thus allowing molecular diagnostics of this key pathway at the single cell level which will be required for future precision medicine.

## 1. Introduction

Advanced cancer is characterized by an increasing degree of tumor cell heterogeneity, resulting in tumor cell populations that display distinct signaling pathways and molecular signatures [1]. This non-uniform distribution of (epi)genetically different tumor-cell subpopulations appears as both temporal and spatial heterogeneity, in the process of disease progression or within disease sites, respectively [2]. Genetic instability is one of the hallmarks of cancer, allowing the rapid evolution of subclones [3]. Pre-existing subclonal populations often show differential responsiveness to therapeutic treatments, thereby contributing to inferior clinical outcomes [4]. Cancer cells often enter into a malicious liaison with cells of both the innate and adaptive immune system, as well as with stroma cells such as tumor-associated macrophages (TAMs), T-cells, or cancer-associated fibroblasts to create a tumor-promoting and immunosuppressive tumor microenvironment [5,6]. A number of studies have found elevated activity of the transcription factor NF-κB in tumor cells which confers tumor cell proliferation, maintenance of cancer stem cells, metastasis, and resistance to therapy [7,8]. On the other hand, NF-κB signaling is often suppressed in tumor-associated immune cells such as TAMs and cytotoxic T-cells, which compromises efficient antitumor immunity [9].

The complexity within the tumor microenvironment and the direct interplay of tumor cells with adjacent healthy tissue cells raises the need to analyze signaling pathways in single cells. While sequencing of single cell genomes or transcriptomes is highly sensitive due to PCR-mediated amplification steps [10,11], single cell proteomic approaches are still in their infancy and are far from covering the entire proteome [12]. These limitations raise the need for alternative methods that allow the analysis of protein/protein complexes or posttranslational modifications of proteins in individual cells. This can now frequently be achieved by in situ proximity ligation assay (PLA) experiments, where two different antibodies bind to neighboring proteins which localize in close proximity, followed by hybridization of secondary antibodies coupled to short ssDNA molecules. The hybridization of connector oligonucleotides and their ligation leads to the generation of templates for consensus sequences which are amplified by a subsequent DNA rolling circle reaction. These multiplied sequences form platforms for the hybridization of fluorescent dyes which can be detected by fluorescence microscopy [13,14]. This methodology not only allows for determining the formation and localization of protein/protein complexes or the analysis of posttranslational modifications, but has been further developed to visualize RNA/protein or RNA/DNA associations [15,16]. PLA has been used to quantify complexes of epidermal growth factor receptor (EGFR) with its adaptor protein growth factor receptor–bound protein 2 (GRB2) in human lung cancer samples, proving that this technique can be used in clinical settings [17]. 

Almost all steps of cancer development can be influenced by aberrant NF-κB activities, as this transcription factor contributes to many direct and indirect steps of oncogenesis and metastasis [18,19]. While the direct effects are due to mutations of members of the NF-κB system itself, indirect effects influence almost all enabling characteristics and hallmarks of cancer [8]. NF-κB is a prototype for an inducible transcription factor system whereby external stimuli lead to the generation of free DNA-binding subunits, which trigger the expression of target genes. The NF-κB family consists of five different NF-κB DNA-binding subunits, which share a N-terminal NF-κB/Rel homology domain (RHD) [20]. This region mediates DNA-binding and dimerization, but also allows interaction with the inhibitory IκB protein that serves to retain NF-κB in the cytosol of unstimulated cells [21,22]. Three of the subunits contain transactivation domains, namely RELA (p65), RELB, and REL (c-Rel), while the subunits p50 and p52 are synthesized as the precursor proteins NF-κB1 (p105) and NF-κB2 (p100) [19,23]. NF-κB activation typically occurs as a cellular response to stress signals such as inflammation, DNA damage, or unfavorable situations. Depending on the cell type and stimulus NF-κB, activation can proceed via the canonical, noncanonical, or the atypical activation pathway [24]. These different pathways finally result in the generation of DNA-binding dimers that mainly serve to trigger the expression of target genes. The canonical NF-κB activation pathway is typically triggered by cytokines such as IL-1α or TNFα, which upon binding to their cognate receptors activate signaling cascades that lead to the transient activation of the so-called IKK (IκB kinase) complex, which is composed of the scaffold protein NEMO and the catalytic subunits IKKα and IKKβ [25,26]. These kinases finally phosphorylate the inhibitory IκBα protein at serines 32 and 36, thereby enabling the subsequent ubiquitination and proteasomal degradation of this IκB protein [27]. This degradation unmasks the nuclear localization sequence contained in NF-κB p65, thus allowing the nuclear import, DNA-binding, and transcriptional activation by NF-κB [28]. NF-κB activation leads to the activation of RNA polymerase II, which is dynamically phosphorylated at the C-terminal domain (CTD) to mediate the recruitment of factors allowing transcription initiation and elongation [29]. This process leads to the induced synthesis of a set of highly unstable mRNAs encoding chemokines (IL-8), cytokines (IL-6), and the negative regulator of NF-κB, inhibitor of NF-κB alpha (IκBα). The re-synthesized IκBα protein serves to terminate the NF-κB response by disrupting NF-κB DNA interactions at chromatin, exporting NF-κB subunits out of the nucleus, and by cytoplasmic retention of p65/p50, while the chemokines and cytokines trigger the inflammatory process [30,31,32,33]. Many mRNAs coding for inflammatory regulators are regulated at the posttranscriptional level by an early phase of stabilization followed at later time points by destabilization [34,35,36]. These mRNA regulatory steps are executed by a number of mechanisms that also include processing (P)-bodies [37]. These ubiquitous mRNPs are found in the cytosol and contain non-translating mRNAs as well as the major components of the 5′-3′ mRNA decay machinery, including the decapping factors DCP1a and EDC4, as well as the exonuclease XRN1 [38,39]. Thus, the NF-κB-driven gene expression is regulated at all levels from de novo transcription to mRNA processing and protein translation.

While many proteins and molecular mechanisms leading to NF-κB have been unraveled by in vitro methods, our knowledge on the intracellular occurrence of protein/protein interactions in intact cells is rather limited. In this study we used mouse embryonic fibroblasts (MEFs) and HeLa cervix carcinoma cells as model systems to study the dynamic regulation of NF-κB under highly standardized and controlled conditions using PLA experiments. These single cell experiments allowed visualizing the IL-1α-dependent dynamic regulation of endogenous p65/IκBα and p65/p50 complexes. PLA was expanded to allow the parallel assessment of p65 nuclear translocation and NF-κB-dependent gene expression in the same cells. This methodology was also used to characterize the interaction of p65 with the P-body component DCP1a, which occurs in a constitutive fashion in the cytosol. Cells with a CRISPR-Cas9-mediated deletion of DCP1a have an impaired nuclear translocation of p65, showing that DCP1a plays a dual role by controlling upstream events in the NF-κB pathway apart from its known decapping activity that mediates posttranscriptional gene regulation of NF-κB target genes.

## 2. Results

### 2.1. IL-1α-Dependent Dynamic Regulation of Endogenous p65/IκBα Complexes in Single Cells

**Figure 1:**
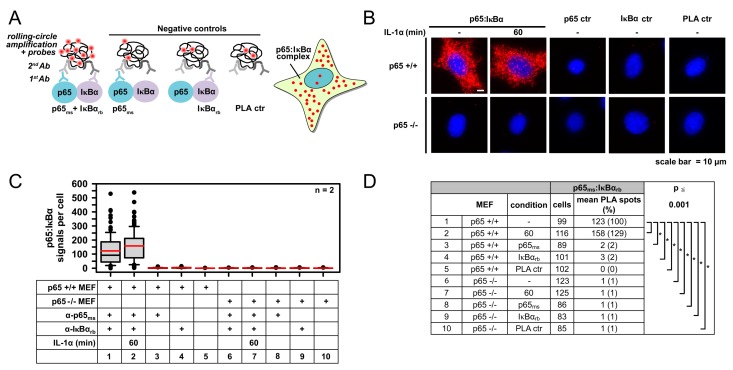
Specificity of proximity ligation assay- (PLA) based detection of p65/IκBα complexes as revealed by the analysis of IL-1α-triggered wild type or p65-deficient mouse embryonic fibroblasts (MEFs). (**A**) Scheme of the PLAs used to determine p65/IκBα heterodimers including three types of negative controls that were used throughout this study to assess background signals. At the end of the PLA procedure, protein complexes were visualized as fluorescent spots by microscopic imaging and signals per cell were counted for quantification. (**B**) Wild type (p65 +/+) or p65-deficient MEFs (p65 −/−) were left untreated or were stimulated with IL-1α (10 ng/mL) for 60 min. Cells were fixed and analyzed by PLA by adding antibodies recognizing p65 (F-6) and IκBα (E130). After the PLA reaction, the nuclei were stained with Hoechst 33342. Representative merged images are displayed. (**C**) PLA signals from individual stained cells were quantified using the Duolink^®^ImageTool software. Box plots visualize the distribution of p65/IκBα complexes (PLA signals) per cell. Data from two independent experiments were pooled. (**D**) The table summarizes the numbers of analyzed cells, PLA signals per cell, relative changes and the significance of changes as obtained by the Mann–Whitney Rank Sum Test (*p* ≤ 0.001).

To visualize the intracellular localization of constitutive p65/IκBα complexes, MEFs were fixed and analyzed for the distribution of these complexes using antibodies specifically detecting both proteins. The PLA set up also included three types of negative controls in which one of the two primary antibodies or both were omitted as schematically shown in (Figure 1A). As an important additional control for the specificity of PLA, these experiments were also performed in p65 knockout MEFs. The standardized analysis of fluorescent cells using the Duolink^®^ImageTool revealed the vast majority of p65/IκBα complexes in the cytosol of unstimulated MEFs and only a small fraction in the nucleus (Figure 1B). Stimulation of cells with IL-1α for 60 min resulted in a slight (but not significant) increase of p65/IκBα complexes, which probably reflects the complete re-synthesis of IκBα after its initial degradation at earlier time points. The number of PLA signals was strongly diminished in the p65 knockout cells or after staining of the MEFs only with single antibodies or omitting both primary antibodies, as revealed by fluorescence microscopy (Figure 1B) and its quantitative and statistical analysis (Figure 1C,D). 

**Figure 2:**
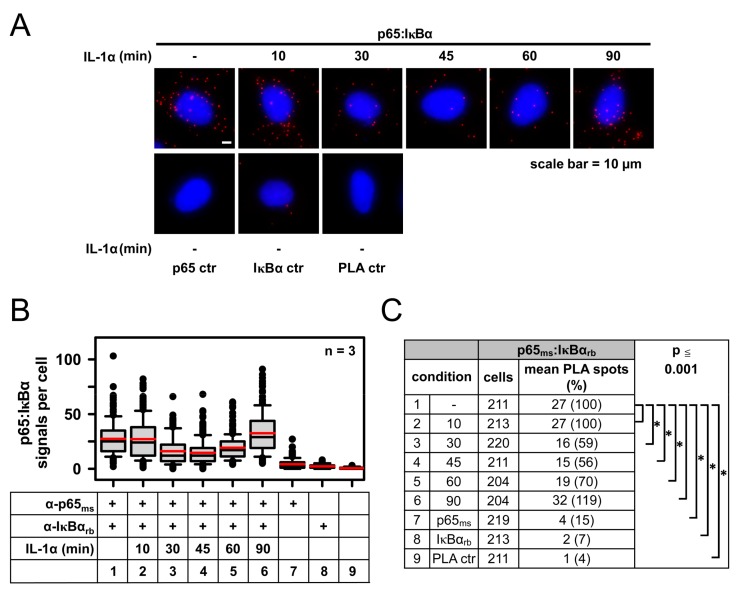
Sensitivity of PLA-based detection of p65/IκBα heterodimers as revealed by the analysis of IL-1α-triggered kinetic changes in complex formation. HeLa cells were left untreated or treated with IL-1α (10 ng/mL) for different time points as indicated. Subsequently cells were fixed and analyzed by PLA using the anti-p65 (F-6) and anti-IκBα (E130) antibodies. As an internal control, antibodies were omitted or used individually. Nuclear DNA was stained with Hoechst 33342. (**A**) Representative merged images are displayed. (**B**,**C**) Data from three independent experiments were pooled. Evaluation and statistical analyses were performed as described for Figure 1. Distribution of PLA signals is shown in (**B**) and the summary and statistics of all relevant data are depicted in (**C**).

These initial experiments ensured that the PLA conditions allowed the highly specific detection of p65/IκBα complexes. To test whether PLA can also capture the dynamic formation and localization of these dimers in physiological set ups, we analyzed their time-resolved formation in IL-1α-stimulated HeLa cells. The latter were stimulated for various periods with IL-1α, followed by the visualization of p65/IκBα complexes using fluorescence microscopy (Figure 2A) and their quantitative and statistical analysis (Figure 2B,C). Administration of IL-1α resulted in a significant decrease of p65/IκBα complexes after 30 min and 45 min, followed by the re-formation of these complexes 90 min after the addition of the stimulus (Figure 2). These kinetic data show that PLA was very sensitive in determining the physiological destruction of IκBα (and hence the decrease of p65/IκBα complexes) and the re-appearance of both, IκBα protein and thus p65/IκBα dimers in tumor cells exposed to inflammatory cytokines. 

**Figure 3:**
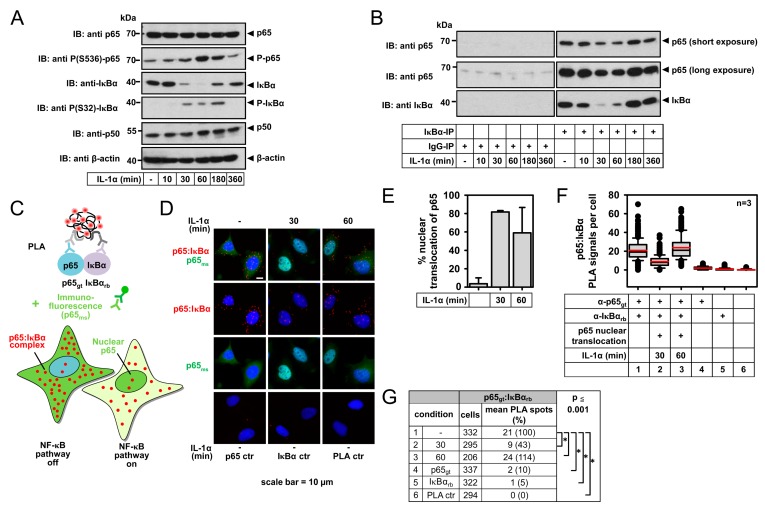
Global functional analysis of p65/IκBα complex formation by co-immunoprecipitation compared to PLA analysis specifically in cells with nuclear translocation of p65. (**A**) HeLa cells were stimulated for the indicated periods with IL-1α (10 ng/mL) as shown. Extracts were prepared and equal amounts of proteins were analyzed by Western blotting for the occurrence and phosphorylation of the indicated proteins with specific antibodies. The position of molecular weight markers is indicated. The experiment is representative for three experiments performed in total. (**B**) The cells were stimulated with IL-1α (10 ng/mL) for the indicated periods and extracts were prepared. While one half of the extract was mixed with antibodies recognizing the IκBα protein, the other half was incubated with control IgG antibodies. Following the addition of True Blot anti rabbit Ig IP agarose beads, the IκBα protein, and the associated proteins were isolated by co-immunoprecipitation, followed by the analysis of proteins by Western blotting as shown. For p65, two different exposure times are shown. (**C**) Scheme of the modified Immuno-PLA procedure that allows discriminating p65/IκBα complex formation in unresponsive cells compared to (neighboring) cells that show nuclear translocation and thus activation of the canonical NF-κB pathway. (**D**) HeLa cells remained untreated or were stimulated for 30 min or 60 min with IL-1α (10 ng/mL) as shown. Cells were fixed and p65/IκBα complexes were revealed by PLA with specific antibodies. This PLA included an additional permeabilization step to improve access of the antibodies to the nuclear compartment. In parallel, the intracellular localization of p65 was analyzed by indirect immunofluorescence using a p65-specific antibody and DyLight 488-coupled secondary anti mouse (ms) antibody. Additionally, nuclear DNA was stained with Hoechst 33342. The cells were analyzed by microscopy, representative merged pictures are shown. (**E**–**G**) Quantification of data from three independent experiments was performed as described in the legend of Figure 1. (**E**) Percentage of cells with nuclear translocation of p65. Bars show means +/− SD. (**F**,**G**) Evaluation and statistical analyses of PLA signals were performed as described for Figure 1. Distribution of PLA signals is shown in (**F**). Note that for the IL-1α-activated situation, PLA signals were specifically counted in cells with nuclear translocation of p65. The summary and statistics of all relevant data are depicted in (**G**).

In parallel, Western blot experiments using extracts from IL-1α-stimulated cells confirmed this pattern of cytokine-induced decay and re-synthesis of IκBα (Figure 3A). Interestingly, the almost complete degradation of IκBα revealed by Western blotting was contrasted by an only incomplete decrease of p65/IκBα complexes detected by PLA (see Figure 2). This finding raises the possibility that the small fraction of IκBα escaping from this degradation is phosphorylated at serines 32/36 and still forms complexes with p65. In fact, co-immunoprecipitation experiments demonstrated that even trace amounts of IκBα remaining after 30 min of IL-1α stimulation still allowed the detection of robust interactions with the endogenous p65 protein (Figure 3B). 

An alternative (and not mutually exclusive) explanation for this observation is the heterogeneity of HeLa cancer cells in responding to IL-1α. Unlike bulk analysis, such as immunoblotting or co-IP using cell lysates, PLA offers the opportunity to analyze p65/IκBα complexes separately in cells with activated NF-κB pathway. To this end, we combined PLA with indirect immunofluorescence by adding a third (anti-p65) antibody, which allows the identification of the population of cells showing an increase of nuclear p65 upon IL-1α stimulation (Figure 3C). As demonstrated by representative images, this analysis facilitates quantifying and distinguishing PLA signals in responding versus non-responding cells within the same specimen (Figure 3D). Quantification of the data confirmed a specific and pronounced IL-1α-mediated transient downregulation of p65/IκBα complexes in cells with strong nuclear accumulation of p65 (Figure 3E–G). This type of Immuno-PLA is therefore highly suited to reveal the different activation states of the canonical NF-κB pathway in heterogeneous tumor cell lines or co-cultures of tumor cells with stroma or immune cells.

### 2.2. Specificity of PLA in Human Cancer Cells and Its Use to Determine p65 Protein Levels

**Figure 4:**
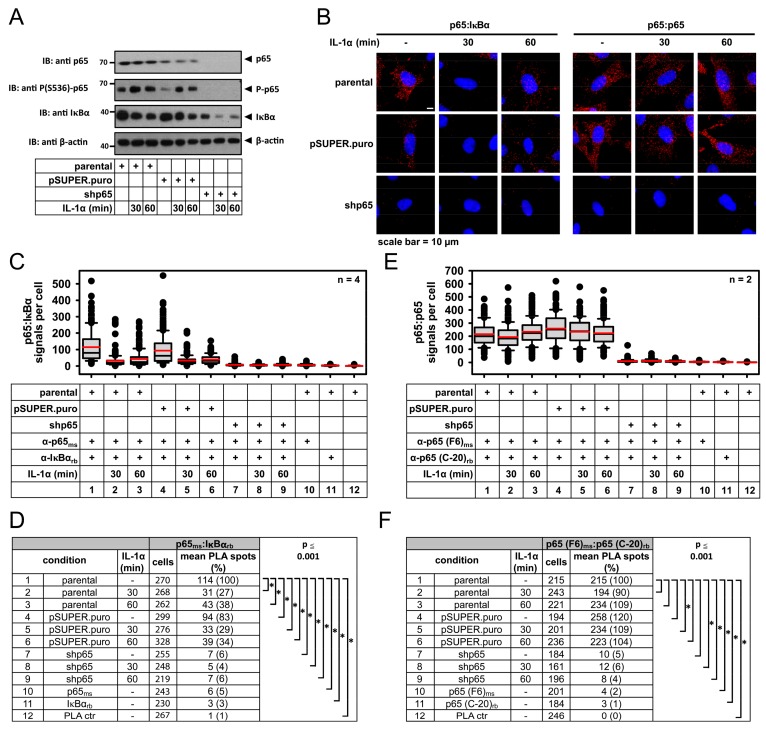
Specificity of PLA in human cancer cells and its use to determine p65 protein levels. HeLa cells were transfected to express a p65-specific shRNA and were treated for three days with puromycin (1 µg/mL) to eliminate the non-transfected cells. These p65 knockdown cells (shp65), cells transfected with empty vector lacking the shRNA-encoding sequence (pSUPER.puro), and the parental non-transfected cells were treated for the indicated periods with IL-1α (10 ng/mL) and the p65/IκBα complex formation or expression of p65 only was revealed by PLA using incubation of fixed cells with antibodies recognizing the N-terminal and C-terminal part of p65. The p65 knockdown samples serve as an additional negative control to ensure the specificity of detected PLA signals. (**A**) A part of the cells was used to determine expression and RNAi-mediated suppression of p65 by immunoblotting. (**B**) Representative merged images of the PLA results assessing p65/IκBα complexes (left graphs) and total p65 protein expression (right graphs). (**C**–**F**) PLA data of cells from four (p65/IκBα PLA) or two (p65/p65 PLA) independent experiments were pooled. Evaluation and statistical analyses were performed as described for Figure 1. Distribution of PLA signals is shown in (**C**,**E**) and the summary and statistics of all relevant data are depicted in (**D**,**F**).

The specificity of the PLA signals detecting p65/IκBα complexes in HeLa cells was further validated by knockdown experiments where shRNA-mediated downregulation of p65, as confirmed by immunoblotting of lysates (Figure 4A), resulted in a strong decrease in the number of detected protein complexes (Figure 4B, lower left images). 

While the p65/IκBα complexes in stimulated cells were mostly cytosolic, it was also interesting to analyze the intracellular localization and expression of p65 only by PLA. A pair of p65-specific antibodies binding to the C-terminal (C-20) and the N-terminal (F-6) parts of the protein allowed detecting p65-specific PLA signals in unstimulated or IL-1α-treated HeLa cells (Figure 4B, upper right images). These signals vanished after shRNA-mediated knockdown of p65 (Figure 4B, lower right images), ensuring the specificity of the employed antibodies. Administration of IL-1α did not change the number of p65 PLA spots (Figure 4B, right images). Quantification and statistical analysis of all these experiments reveal that PLA is also very sensitive and specific for determining protein levels and subcellular distribution of p65 (Figure 4C–F).

### 2.3. Detection of IL-1α-Regulated Active Nuclear NF-κB Complexes by PLA

The nucleus contains the densely packed high-molecular chromatin fraction and ribonucleoprotein particles, thus creating an environment with decreased accessibility to antibodies [40]. To circumvent these problems, we used a modified Duolink protocol which employs an additional permeabilization step of the nuclear membrane (see methods for details) to visualize the localization and activity of NF-κB p50/p65 heterodimers in the nucleus. 

**Figure 5:**
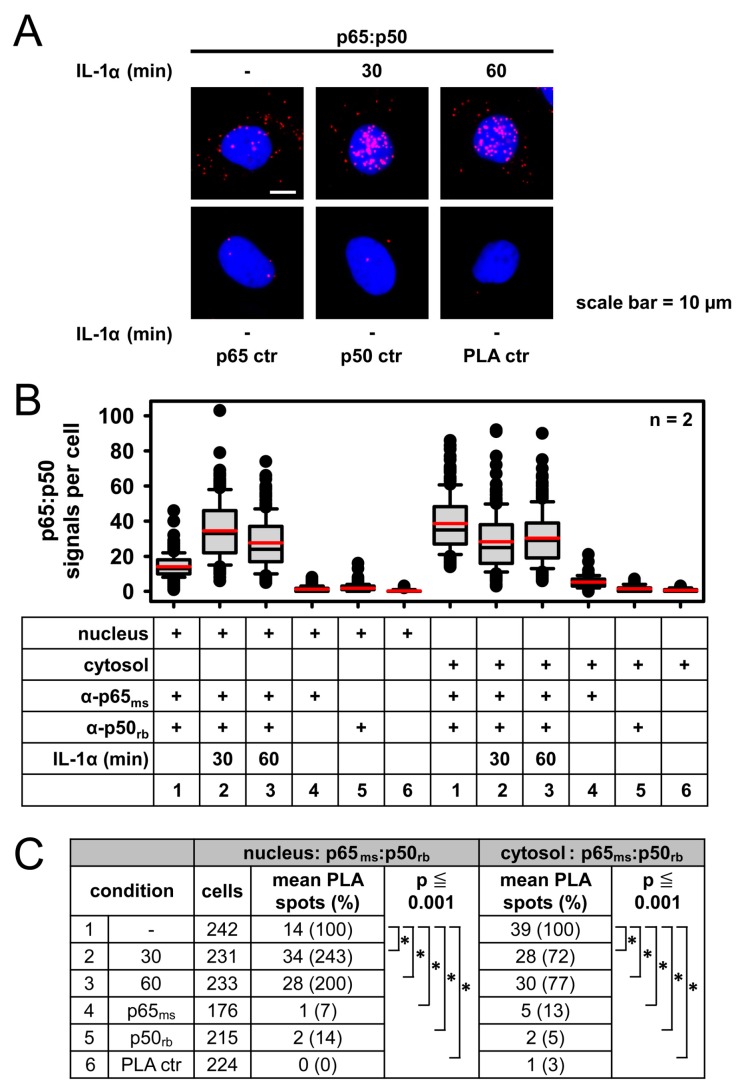
PLA-based analysis of IL-1α-induced translocation of p65/p50 heterodimers to study NF-κB functions in the nucleus. (**A**) HeLa cells were left untreated or stimulated with IL-1α (10 ng/mL) as shown. Cells were permeabilized using an adapted PLA protocol to increase the access of antibodies to the nuclear fraction, followed by PLAs using antibodies recognizing p50 and p65, respectively. Representative cells are displayed: Nuclei were visualized by Hoechst 33342 staining. (**B**,**C**) PLA data of cells from two independent experiments were pooled. Evaluation and statistical analyses were performed as described for Figure 1. Distribution of PLA signals is shown in (**B**) and the summary and statistics of all relevant data are depicted in (**C**).

Subcellular fractionation, chromatin immunoprecipitation sequencing (ChIP-seq), and ChIP-qPCR experiments for NF-κB p50 and p65 subunits revealed the IL-1α-inducible association of both NF-κB subunits to enhancer and promoter regions, but also to exons and intergenic regions [41,42]. In unstimulated HeLa cells, PLAs detected the p50/p65 dimers primarily in the cytosol, whereas the addition of IL-1α resulted in a decrease of cytosolic and a parallel increase of nuclear p50/p65 complexes, demonstrating the suitability of the PLA approach for the visualization of nuclear and functionally active NF-κB complexes (Figure 5A–C). 

### 2.4. Combining PLA with Single Cell Gene Expression Analysis Using Single Molecule RNA Fluorescence in Situ Hybridization (smRNA-FISH)

**Figure 6:**
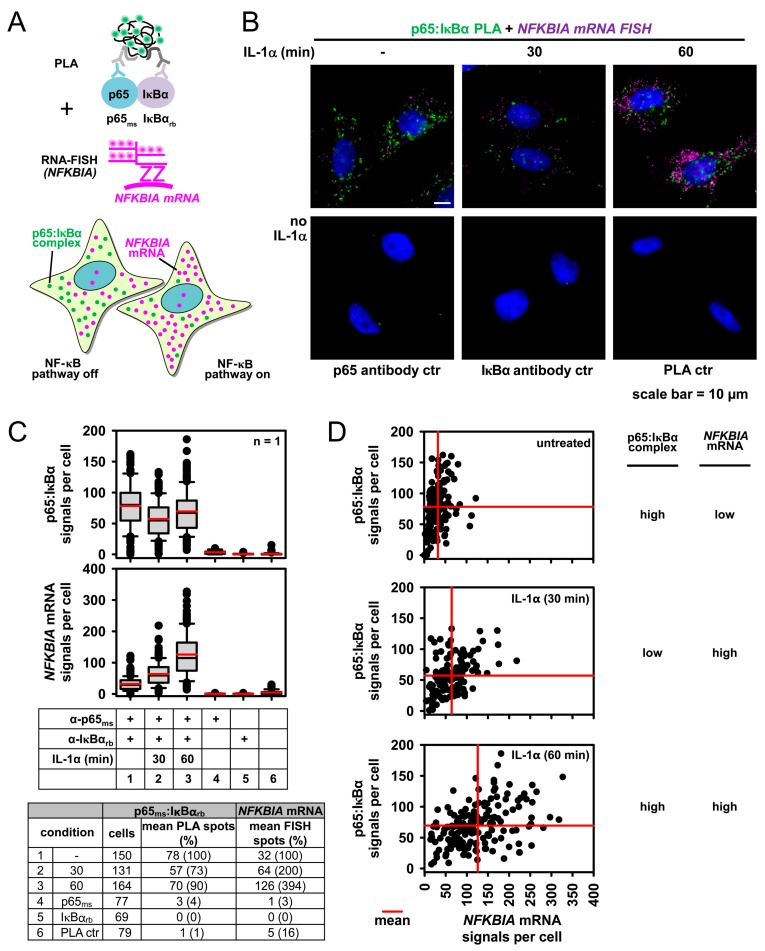
PLA combined with RNA-FISH of the IL-1α target gene *NFKBIA* to study downstream NF-κB functions in the nucleus. (**A**) Scheme of the modified PLA procedure coupled to single molecule (sm)RNA-FISH that allows to discriminate p65/IκBα complex formation in unresponsive cells compared to (neighbouring) cells with IL-1α-induced mRNA expression of the *NFKBIA* gene that encodes the IκBα protein. (**B**) HeLa cells remained untreated or were stimulated for 30 or 60 min with IL-1α (10 ng/mL) as shown. Cells were fixed and p65/IκBα complexes were revealed by PLA with specific antibodies followed by *NFKBIA* smRNA-FISH. PLA signals appear in green, *NFKBIA* FISH signals in pink and nuclei in blue (stained with Hoechst 33342). The cells were analyzed by fluorescence microscopy, representative merged pictures and PLA negative controls (from untreated cells) are shown. (**C**) Both, PLA and smRNA-FISH signals were counted by the Duolink^®^ImageTool. The box plots show the distribution of PLA or smRNA-FISH signals. The table summarizes all relevant single cell data. (**D**) For each cell the number of p65/IκBα complexes was plotted against the *NFKBIA* smRNA-FISH signals. Data from untreated and IL-1α-stimulated conditions are depicted as separate scatter plots, red lines indicate mean signals.

We then extended the PLA approach for the measurement of the NF-κB function by combining PLA with single cell mRNA expression analysis. For this we added smRNA-FISH probes specific for the *NFKBIA* mRNA, which encodes the IκBα protein to the PLA reaction. The principle and expected outcome of this assay is schematically shown in Figure 6A and examples of images are shown in Figure 6B. At the peak of NF-κB pathway activation in the cytoplasm, p65/IκBα dimers declined. At the same time and as a consequence of nuclear translocation of liberated p65, the activation of canonical NF-κB target genes started to increase. Consequently, there are reduced p65/IκBα signals after 30 min of IL-1α stimulation, whereas the number of *NFKBIA* mRNAs was upregulated and further increased at 60 min after IL-1α treatment due to ongoing transcription (Figure 6B). This pattern is also seen by pooling the data from all analyzed cells (Figure 6C). However, this approach also allows tracing the correlation of p65/IκBα complex formation with downstream activation of the NF-κB target gene for each individual cell. Scatter plots show the intercellular variation compared to the mean PLA or RNA-FISH signals (red lines) of the cell cultures in each condition (Figure 6D). We conclude that this type of dual NF-κB pathway analysis is a powerful tool to identify the potential desynchronization or uncoupling of the cytosolic NF-κB pathway from the nuclear gene response at the single cell level. 

### 2.5. DCP1a Constitutively Associates with p65 to Indirectly Control Its IL-1α-Triggered Nuclear Import

**Figure 7:**
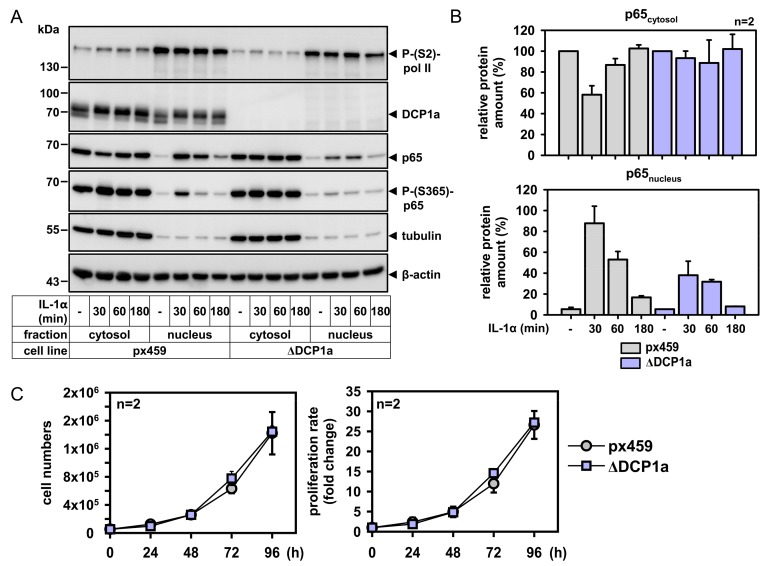
DCP1a is required for efficient nuclear translocation of p65 NF-κB. (**A**) HeLa cell lines with CRISPR/Cas9-mediated suppression of DCP1a (ΔDCP1a) or cells stably transfected with the control vector (px459) were incubated for the indicated times with IL-1α (10 ng/mL) or were left untreated. Cytosolic and nuclear extracts were prepared and the presence of p65 and DCP1a proteins was examined by immunoblotting. Anti-tubulin, β-actin, and P(S2)-RNA polymerase II antibodies were used to control for purity of the fractions and for equal loading. Shown is one out of two independent experiments with compatible results. (**B**) Shows relative quantification of mean p65 protein amounts +/− SD from two independent experiments. (**C**) The same cells were seeded at two different densities and cell numbers were assessed at the indicated time points. The graphs show cell numbers and corresponding fold changes.

It was then interesting to use the PLA approach for the analysis of new or poorly characterized NF-κB interaction partners. We have previously found an interaction between p65 and the P-body component DCP1a in co-immunoprecipitation experiments using overexpressed proteins [43]. Additionally, in this work a functional link between both proteins was established by showing that overexpressed DCP1a reduced the activities of a NF-κB-driven reporter gene [43]. Here, subcellular fractionation experiments showed a reduction of nuclear p65 after IL-1α treatment in cells depleted for endogenous DCP1a by CRISPR-Cas9, revealing an additional positive (and upstream) role of DCP1a in regulation of NF-κB (Figure 7A,B). As NF-κB can modulate the cell cycle [44], we determined proliferation rates of these cells. However, DCP1a-deficient cells did normally proliferate over a time course of 96 h, suggesting that the role of DCP1a in NF-κB signaling is unrelated to cell cycle control (Figure 7C). 

**Figure 8:**
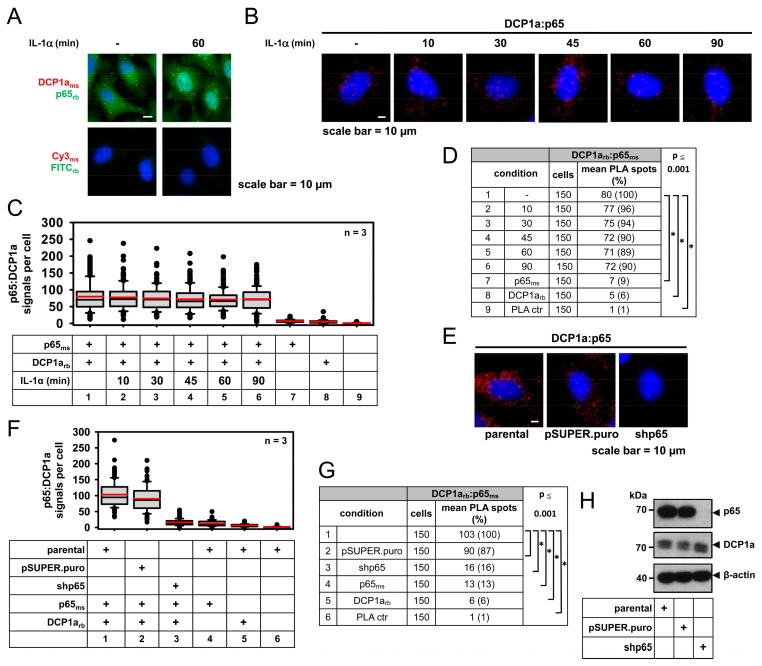
PLA for the validation of new p65 interactors: Analysis of endogenous NF-κB p65/DCP1a complexes. (**A**) Double-immunofluorescence analysis of DCP1 and p65 in HeLa cells stably transfected with pSUPER.puro. (**B**–**D**) PLAs were used to determine the interaction of endogenous DCP1a with p65 NF-κB in HeLa cells before and after stimulation with IL-1α (10 ng/mL) at different times. The specificity of the obtained PLA signals was validated by omitting one or both primary antibodies. (**E**–**G**) Additionally, specificity of PLA was assessed in cells transiently transfected for 24 h with a p65 shRNA construct or with empty vector (pSUPER.puro) control. Representative images, the summary of PLA quantification and statistical tests are shown. (**E**–**G**) Data were pooled from three independent experiments (50 cells each). Distribution of PLA signals and the summary and statistics of all relevant data are depicted graphically and as tables as before. (**H**) The p65 knockdown was also confirmed in cell extracts by immunoblotting.

Immunofluorescence analysis confirmed the mainly cytosolic and P-body localization of DCP1a (Figure 8A). We therefore used PLA to prove that endogenous DCP1a indeed interacts with p65. A quantitative analysis of the PLA spots revealed that the p65/DCP1a complexes occurred in a constitutive manner and were not significantly affected by IL-1α treatment between 10 min to 90 min of stimulation (Figure 8B–D).

The specificity of the observed interactions was ensured in control experiments where the number of PLA signals was strongly diminished after a knockdown of p65 or after staining with single antibodies (Figure 8E–G). HeLa cells showed the occurrence of PLA spots representing the interaction sites between DCP1a and p65 largely in the cytosol and these signals were lost upon depletion of p65, which was validated by immunoblotting of lysates (Figure 8H). 

Together, these data demonstrate how protein/protein interactions for new binding partners of NF-κB components can be validated at the endogenous protein level by specific and sensitive PLA. In the case of DCP1a, they show that the interaction between the endogenous DCP1a and p65 proteins occurred constitutively in the cytosol and that DCP1 is a critical regulator of p65 nuclear translocation.

## 3. Discussion

The heterogeneity of many advanced solid tumors in combination with the intricate interactions with tumor surrounding cells raises the need to analyze specific protein complexes, their pathway-dependent regulation, and their downstream consequences for gene expression at the single cell level. These approaches allow the complex analysis of signaling cascades conferring tumor adaptations to enable precision medicine approaches for the specific targeting of cancer cells. The analysis of protein functions in individual cells can be measured by a variety of proximity-dependent assays, however, most of these approaches require the prior transfection or genetic manipulation of the cells [45]. The intrinsic advantage of PLAs and all their variations is the fact that even trace amounts of fixed cells are sufficient to enable the analysis of signaling events using antibodies without any need for prior interference with the cells. Therefore, PLAs are increasingly used for the analysis of signaling in tumor cells to improve the accuracy of diagnosis [14,46]. These assays can be also performed with non-adherent cells such as lymphomas and they can even visualize interactions of proteins contained on the surface of two different cells [47,48]. The constant development of PLA protocols together with progress in the generation of high quality antibodies, standardized protocols, automated picture analysis, and a pipeline of bioinformatics analysis tools will enable a quantitative, reliable, and comparable analysis of dynamic signaling events in single cells and further improve molecular diagnostics of tumors. 

Although previous studies used the PLA approach for the validation of protein/protein interactions in the NF-κB system [49,50], a systematic study investigating the dynamic regulation of NF-κB at all levels of gene expression has not yet been performed. This study shows the suitability of PLAs for the quantitative and time-resolved analysis of IL-1α-induced dynamic NF-κB regulation, unravelling further aspects of NF-κB signaling. While Western blotting detects almost complete IL-1α-inducible degradation of IκBα and a strong phosphorylation of the residual amounts of IκBα escaping from proteasomal degradation, PLAs show that these remaining IκBα proteins preferentially interact with p65 in the cytosol. This finding is compatible with the idea that phosphorylated IκBα shows a high affinity to cytosolic p65. A minority of p65/IκBα complexes occurs in the nucleus, where IκBα might dissociate NF-κB from its cognate DNA and promote active transport of NF-κB to the cytoplasm [32,51].

Two types of adapted PLA protocols allow further dissection of the NF-κB signaling cascade in individual cells. Immuno-PLA and PLA-smRNA-FISH can be used for assessing the formation of active (or inactive) signaling complexes in relation to nuclear translocation and downstream gene activation of NF-κB components. We expect that the combined application of these approaches in heterogeneous tumor cell lines, co-cultures of tumor cells with stroma or immune cells or in the tumor tissue itself will provide important insight into the cell-type specific activation state of the NF-κB pathway in situ. 

Our results also identified the P-body component DCP1a as a constitutive p65 interaction partner which is required for efficient IL-1α-induced p65 nuclear translocation. This effect is probably due to the incomplete IL-1α-inducible degradation of IκBα in the absence of DCP1a, but could potentially also involve additional mechanisms. The P-body protein DCP1a plays an important role for the decapping and decay of inflammatory mRNAs, thus serving as a critical component of posttranscriptional gene regulation [52]. This study revealed its additional function in an upstream step leading to NF-κB activation. Such a dual role for upstream events and posttranscriptional gene regulation has also been described for TRAF6, which functions as an ubiquitin E3 ligase mediating the formation of K63-branched chains. This TRAF6-mediated ability is required for TRAF6-dependent activation of the IKK complex and also for the formation of P-bodies [52,53]. The activity of one protein at multiple levels of gene expression might serve to ensure the tight cross-regulation between all levels of gene expression, which is considered to be an integrated process coordinated by many regulatory loops [54]. In this context, it is interesting to note that EDC4, an integral component of P-bodies, has been implicated in IKK signaling as well as in breast cancer [36,55]. EDC4 serves as a scaffold for DCP2, DCP1a, and the 5′ exonuclease XRN1 in the decapping pathway [56]. Thus, emerging evidence suggests a connection between P-body components, NF-κB signaling, and cancer and our study provides a set of tools and approaches to study these events in individual (tumor) cells. 

## 4. Materials and Methods

### 4.1. Cell Culture

HeLa cells [57] and wild type or p65/*RELA*-deficient murine embryonic fibroblasts, kindly provided by Ezra Burstein [58,59], were maintained in Dulbecco’s modified Eagle’s medium (DMEM), complemented with 10% fetal calf or filtrated bovine serum (FCS or FBS), 2 mM of L-glutamine, 100 U/mL of penicillin, and 100 µg/mL of streptomycin. Stable pools of DCP1a-depleted cells generated by transfection of px459-based CRISPR/Cas9 constructs were selected and maintained in puromycin (1 µg/mL). Prior to all experiments, puromycin was omitted for 24 h. IL-1α (10 ng/mL) was added directly to the cell culture medium. Cells were counted in a Neubauer chamber. The viability of vector control and ΔDCP1a cells (Figure 7) was 100% as assessed by trypan blue staining. 

### 4.2. Plasmids, Transient, or Stable Transfections

The following plasmid was obtained commercially: pSpCas9(BB)-2A-Puro (px459, Addgene (#48139). pSUPER.puro has been described [41]. The following vectors were cloned: px459-ΔDCP1a (containing sg3-DCP1a, (se) 5′-CACCGTGGGCAGGAGATGAGCCTAG-3′, (as) 5′-AAACCTAGGCTCATCTCCTGCCCAC-3′), pSuper.Puro shp65 (containing shRNA (se) 5′-GATCCCCGGATTGAGGAGAAACGTAAttcaagagaTTACGTTTCTCCTCAATCCTTTTTGGAAA-3′, (as) 5′-AGCTTTTCCAAAAAGGATTGAGGAGAAACGTAAtctcttgaaTTACGTTTCTCCTCAATCCGGG-3′. 

For transient transfection 1,200,000 cells were seeded in 100 mm dishes. The transfection approach for calcium-phosphate includes 30 µg of plasmid-DNA, 1.359 mL of dH_2_O, 1.51 mL of 2× HEBS, and 0.19 mL of ice-cold CaCl_2_. After 10 min of incubation at room temperature, the transfection mixture was added dropwise to the cells and incubated for 5 h at 37 °C. For yielding higher transfection efficiency, a glycerol shock was performed for 3 min at room temperature. Subsequently the cells were washed two times with PBS following the addition of fresh medium. After 24 h, the cells were washed with PBS+EDTA (0.4 g/L), trysinized, and seeded for 3 days of selection in puromycin (1 µg/mL). For single cell analysis, cells were seeded at 6900 (pSUPER.puro) or 7200 (shp65) cells per slot in µ-slides VI (Ibidi) and for Western blot analyzes, cells were seeded at 180,000 (pSUPER.puro) or 200,000 (shp65) per 60 mm dish, respectively. 

For stable sgRNA transfections, cells were transfected by the calcium-phosphate method and pools of cells were selected in complete medium with 1 µg/mL of puromycin.

### 4.3. (Immuno)-Proximity Ligation Assays

We performed in situ PLA using the Duolink kit (Merck, #DUO92007, Darmstadt, Germany). Based on the manufacturers’ protocol, we established and optimized a standardized protocol to perform PLA in the µ-slide system from Ibidi (Graefelfing, Germany). A total of 9000 cells were seeded for 24 h in µ-slides VI (Ibidi). After washing, cells were fixed with 4% paraformaldehyde in PBS (Santa Cruz, #281692, Dallas, TX, USA) for 5 min, permeabilized with Hank’s BSS (HBSS, PAN-Biotech, #P04-32505, Aidenbach, Germany) containing 0.1% or 0.005% saponin (Merck, Darmstadt, Germany) for 2 × 5 min and blocked with the blocking solution provided in the Duolink kit for 30 min at 37 °C followed by incubation for 1 h at 37°C in a humidity chamber with primary rabbit (rb), mouse (ms), and goat (gt) antibodies diluted in the Duolink antibody diluent. 

Goat (gt) anti-p65 (Bethyl Laboratories #A303-945A, Montgomery, TX, USA) was diluted 1:1,000, rb anti-p65 (C-20, Santa Cruz, #sc-372, Dallas, TX, USA) was diluted 1:200, all remaining antibodies: rb anti-DCP1a (Abcam, #ab47811, Cambridge, UK), ms anti-p65 (F-6, Santa Cruz, #sc-8008, Dallas, TX, USA), rb anti-p50 (Abcam, #ab7971, Cambridge, UK), and rb anti-IκBa (Abcam,# ab32518, Cambridge, UK) were diluted 1:100.

After washing three times for 5 min in wash buffer A, cells were incubated with the PLA probes anti goat-Minus (Merck, #DUO92006-100RXN, Darmstadt, Germany) or anti-mouse Minus (Merck, #DUO92004-100RXN, Darmstadt, Germany) and anti-rabbit Plus (Merck, #DUO92002-100RXN, Darmstadt, Germany), diluted 1:5 in the Duolink antibody diluent, for 1 h at 37 °C in a humidity chamber. 

For the additional detection of p65 protein, an anti-mouse DyLight488-conjugated secondary antibody (Biozol Diagnostica, #DkxMu-003D488NHSX, Eching, Germany, 1:100) was added to the PLA probes diluted in Duolink antibody diluent. Cells were washed three times for 5 min in wash buffer A and ligation (30 min) and amplification (100 min) were performed with the Duolink in situ detection reagents orange at 37 °C in a humidity chamber. After the final washing steps in wash buffer B (3 × 5 min), nuclei were counterstained with Hoechst 33342 (Thermo Fisher Scientific, Waltham, MA, USA). For controls, incubations with only one primary antibody or omission of both primary antibodies were performed in parallel. 

For the analysis of nuclear p65/p50 complexes as well as the detection of p65 by Immuno-PLA, an additional lysis step was introduced as follows. Subsequent to stimulation with IL-1α (10 ng/mL), the cells were washed twice within 5 min with PBS and fixed with 4% paraformaldehyde in PBS (Santa Cruz, #281692, Dallas, TX, USA) for 10 min. To obtain a stabilizing condition, DNA cells were incubated in 0.1 M of Tris-Cl (pH 7.4) for 10 min and permeabilized with 0.005% saponin/0.1% Triton-X in PBS for 10 min. After washing it twice with PBS, the cells were incubated in 40% glycerol in PBS overnight to obtain a cryoprotection. After 12 h of incubation, 3 freeze and thaw cycles (1 min) were used to open the chromatin structure. Following 2 × 5 min washing with PBS, the cells were blocked for PLA. The remaining PLA procedure steps were performed as described above.

Imaging was performed on a Leica DMIRE2 or DMi8 fluorescence microscope using filter cubes suited for Cy3 (excitation 560/40 and emission 630/75) and DyLight488 (excitation 480/40 and emission 527/30). Captured images were analyzed with the Leica FW4000 Fluorescence Workstation software (version 1.2.1) or Leica LASX software (version 1.5.1.13187), respectively. Quantification of PLA spots was performed using the Duolink Image Tool (version 1.0.1.2) from Olink Bioscience usually with the following settings for the signal channel red: Nuclei size (px):40-80; cytoplasm size (px):100-150, signal threshold: 55-250, signal size (px):5-7.

### 4.4. PLA Coupled to Single Molecule RNA-Fluorescence in Situ Hybridization (PLA-smRNA-FISH)

For detection of *NFKBIA* transcripts, the Affymetrix FISH kit QuantiGene^®^ ViewRNA ISH Cell Assay (Thermo Fisher Scientific, QVC0001, Waltham, MA, USA) was used in combination with specific branched-probe sets against *NFKBIA* (VA6-17971) according to the manufacturer’s instruction. This technique was performed subsequently to PLA as follows. 

A total of 9000 cells were seeded for 24 h in µ-slides VI (Ibidi), stimulated with IL-1α (10 ng/mL), and washed twice with HBSS within 5 min. Subsequently, cells were fixed with 4% (w/v) paraformaldehyde in PBS (Santa Cruz, #281692, Dallas, TX, USA) at 4 °C, overnight. Following permeabilization with 0.005% saponin in HBSS for 5 min twice, the PLA was performed with the Duolink in situ detection reagents green (Merck, #DUO92014, Darmstadt, Germany) as described above. Then, cells were washed three times with buffer B and twice for 1 min with PBS-Tween (1:1000) at room temperature to maintain the membrane permabilization. The hybridization with the probe sets (diluted 1:100) was performed at room temperature in a humidity chamber overnight. For the detection of labeled mRNAs preamplifier mix, amplifier mix, and label probe mix (diluted 1:30, respectively) were sequentially added, each incubated at 40 °C for 30 min. Cells were washed twice for 2 min and once for 10 min with the wash buffer. 

Fluorescence analyses were performed using the inverse microscope DMi8 (Leica) using filter cubes suited for DyLight488 (excitation 480/40 and emission 527/30), Cy5 (excitation 620/60 and emission 700/75), and Leica LASX software (version 1.5.1.13187). Quantification of mRNA transcripts was performed using the Duolink Image Tool (version 1.0.1.2) from Olink Bioscience. 

### 4.5. Software Used, Quantification, and Statistical Analyses

Statistics (Mann–Whitney Rank Sum Test) and quantifications were calculated using SigmaPlot11 or MSEXCEL2010. In all box plots, the boundary of the box closest to zero indicates the 25th percentile, a black line within the box marks the median, a red line marks the mean, and the boundary of the box farthest from zero indicates the 75th percentile. Whiskers (error bars) above and below the box indicate the 90th and 10th percentiles. Dots represent outlying points. 

### 4.6. Cell Lysis, Western Blotting, and Co-Immunoprecipitation Experiments

For whole cell extracts, cells were lysed in a Triton cell lysis buffer (10 mM Tris, pH 7.05, 30 mM NaPPi, 50 mM NaCl, 1% Triton X-100, 2 mM Na_3_VO_4_, 50 mM NaF, 20 mM ß-glycerophosphate and freshly added 0.5 mM PMSF, 2.5µg/mL leupeptin, 1.0 µg/mL pepstatin, 1 µM microcystin). Cells were incubated for 15 min on ice and lysates were cleared by centrifugation for 15 min at 14,000× *g*. Protein concentration of supernatants (corresponding to total lysates) was determined by Bradford assay.

For preparation of nuclear protein extracts (Figure 7A), cells were harvested after washing in cold PBS and collected by centrifugation. The pellet was suspended in extraction buffer A (10 mM HEPES pH 7.9, 10 mM KCl, 1.5 mM MgCl_2_, 300 µM Na_3_VO_4_, 20 mM β-glycerophosphate, 10 µM E64, 5 mM DTT, 2.5 µg/mL leupeptin, 1 µM pepstatin, 300 µM PMSF, 1 µM microcystin) and centrifuged for 5 min at 800× *g* at 4 °C) and resuspended again in extraction buffer A containing NP-40 with a final concentration of 0.1%. After incubation on ice for 10 min, samples were centrifuged (5 min, 15.800× *g*, 4 °C) and the supernatants (corresponding to cytoplasmic extracts) were collected. Nuclear extracts were prepared by resuspending pellets in extraction buffer B (20 mM HEPES pH 7.9, 420 mM NaCl, 1.5mM MgCl_2_, 200 µM EDTA, 300 µM Na_3_VO_4_, 20 mM β-glycerophosphate, 10 µM E64, 5 mM DTT, 2.5 µg/mL leupeptin, 1 µM pepstatin, 300 µM PMSF, 1 µM microcystin, 25% glycerol) followed by mixing, incubating on ice for 30 min, and centrifugation (5 min, 15.800× *g*, 4 °C). The supernatants correspond to nuclear extracts. 

For co-immunoprecipitation experiments (Figure 3A,B), cells were harvested after washing in PBS and collected by centrifugation. The pellet was directly lysed in a cell lysis buffer (50 mM HEPES pH 7.4, 50 mM NaCl, 1% Tween20, 2.5 mM EGTA, 1 mM EDTA, 1 mM NaF, 10 mM β-glycerophosphate, 0.1 mM Na_3_VO_4_, 1 mM PMSF, 1 mM DTT, 1 × protease inhibitor cocktail (from Roche, Basel, Switzerland), 1 µM microcystin) and incubated for 20 min on ice. The DNA was sheared by three sonication steps of 20 s and lysates were cleared by ultracentrifugation for 20 min at 100.000× *g* and 4 °C. IκBα was immunoprecipitated from 1 mg of precleared cell extracts using 0.085 µg anti IκBα antibody (Cell Signaling, #9242S, Cambridge, UK) or 1 µg of normal IgG (Santa Cruz, #sc-2027, Dallas, Texas, USA) coupled for 2 h to 15 µl TrueBlot ^®^ anti-rabbit Ig IP agarose beads (Rockland, #00-8800-25, Limerick, PA, USA). For precipitation, 1 mg of cell extract was incubated with pre-coupled beads in a total volume of 400 µl of lysis buffer for 2 h at 4 °C.

After rotating for 2 h at 4 °C, the supernatant was discarded and the beads were washed four times with a high salt wash buffer (50 mM HEPES pH 7.4, 450 mM NaCl, 1% Tween20, 2.5 mM EGTA, 1 mM EDTA, 1 mM NaF, 10 mM β-glycerophosphate, 0.1 mM Na_3_VO_4_, 1 mM PMSF, 1 mM DTT, 1x protease inhibitor cocktail from Roche, Basel, Switzerland). The precipitated proteins were eluted by boiling in 2x Roti-Load for 10 min before analyzing by Western blotting.

Cell lysates, subcellular fractions, or immunoprecipitates were subjected to SDS-PAGE on 7–12.5% gels and immunoblotting was performed essentially as described [60]. Proteins were separated on SDS-PAGE and electrophoretically transferred to PVDF membranes (Carl Roth, Roti-PVDF (0,45 µm), Karlsruhe, Germany). After blocking with 5% dried milk in Tris-HCl-buffered saline/0.05% Tween (TBST) for 1 h, membranes were incubated for 12–24 h with primary antibodies, washed in TBST, and incubated for 1–2 h with the peroxidase-coupled secondary antibody. Proteins were detected by using enhanced chemiluminescence (ECL) systems from Millipore or GE Healthcare. Images were acquired and quantified using a Kodak Image Station 440 CF and the software Kodak 1D, 3.6, or the ChemiDoc TouchImaging System (BioRad, Hercules, CA, USA) and the software ImageLab V_5.2.1 (Bio-Rad), or X-ray films and the software ImageJ.

All immunoblot data are provided as Supplementary Materials. 

### 4.7. Antibodies and Reagents

Human recombinant IL-1α was a kind gift from Jeremy Saklatvala, Oxford, UK, or was prepared in our laboratory as described [43]. The following inhibitors were used: Leupeptin hemisulfate (Carl Roth, #CN33.2, Karlsruhe, Germany), microcystin (Enzo Life Sciences, #ALX-350-012-M001, Farmingdale, NY, USA), pepstatin A (Applichem, #A2205, Darmstadt, Germany), PMSF (Thermo Fisher Scientific, #P-7626, Waltham, MA, USA). Pepstatin A, PMSF and microcystin were dissolved in ethanol and leupeptin as well as the protease inhibitor cocktail tablet in dH2O. Other reagents were from Sigma-Aldrich or Thermo Fisher Scientific and were of analytical grade or better. 

Primary antibodies against the following proteins or peptides were used: Anti β-actin (Santa Cruz, #sc-4778, Dallas, TX, USA), anti DCP1a (rb, Abcam #ab47881, Cambridge, UK), anti DCP1a (ms, Abnova, #H00055802-M06, Taipei, Taiwan), anti IκBα (rb, Cell Signaling, #9242, Cambridge, UK), anti NFκB p65 (rb, Santa Cruz, #sc-8008, Dallas, TX, USA), anti NFκB p65 (rb, Santa Cruz, #sc-372, Dallas, TX, USA), anti P(S2)-RNA polymerase II (rb, Abcam, #ab5095, Cambridge, UK), anti RNA polymerase II (ms, Merck Millipore, #17-620, Darmstadt, Germany), anti P(S536)-p65 NFκB (rb, Cell Signaling, #3033, Cambridge, UK), anti (PS32)-IκBα (rb, Cell Signaling, #2859, Cambridge, UK), and anti tubulin (ms, Santa Cruz, #sc-8035, Dallas, TX, USA). 

Secondary antibodies were: Cy3-coupled anti mouse (ms) IgG (dk, Merck Millipore, #AP192C, Darmstadt, Germany), DyLight488-coupled anti mouse (ms) IgG (dk, Biozol Diagnostica, #DkxMu-003D488NHSX, Eching, Germany), FITC coupled anti rabbit (rb) IgG (dk, Merck, #F0257, Darmstadt, Germany), HRP-coupled anti mouse (ms) IgG (gt, Agilent, #P0447, Santa Clara, CA, USA), HRP-coupled anti rabbit (rb) IgG (gt, Agilent, #P0448, Santa Clara, CA, USA), and HRP-coupled anti rabbit (rb) IgG (gt, Thermo Fisher Scientific, #31460, Waltham, MA, USA).

## 5. Conclusions

**Figure 9:**
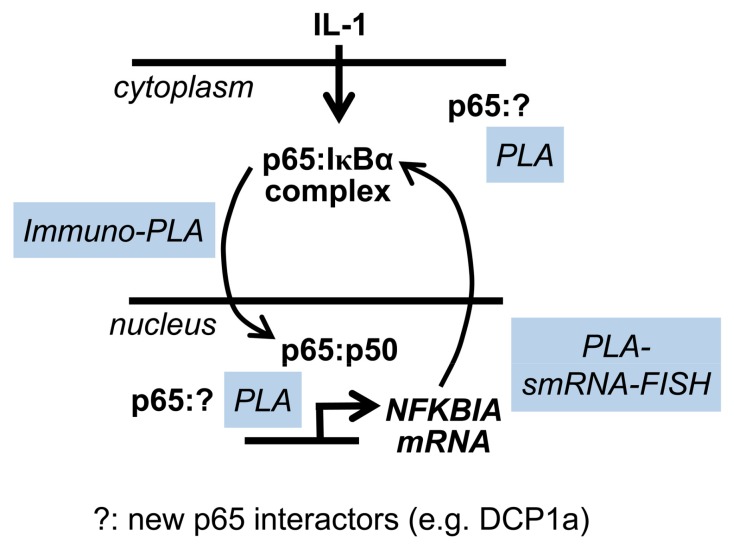
Summary: PLA to study multiple levels of NF-κB activation in individual tumor cells at the single cell level.

This study demonstrated how the specific and sensitive PLA approach can be used to quantify the dynamic IL-1α-regulated interaction of the DNA-binding subunit p65 with (i) its suppressor IκBα, (ii) the nuclear NF-κB p50 subunit, and (iii) the novel interaction partner DCP1a in single cells. By combining PLA approaches with immunostaining for p65 or smRNA-FISH, further aspects of NF-κB function could be integrated in the analysis. As schematically summarized in Figure 9, these high resolution approaches are therefore well suited to diagnose the activation status of the NF-κB system at multiple levels in complex tumor samples.

## Supplementary Materials

The following are available online at https://www.mdpi.com/2072-6694/11/8/1199/s1, Original blots data.
