# Supplementary material for: Single-Cell Analysis of Multiple Steps of Dynamic NF-κB Regulation in Interleukin-1α-Triggered Tumor Cells Using Proximity Ligation Assays
Source: Cancers (Basel). 2019 Aug 16;11(8):1199. doi: 10.3390/cancers11081199 (PMC6721548; doi:10.3390/cancers11081199)
Supplement: Supplementary file 1 [file cancers-11-01199-s001.pdf]

Figure 3A

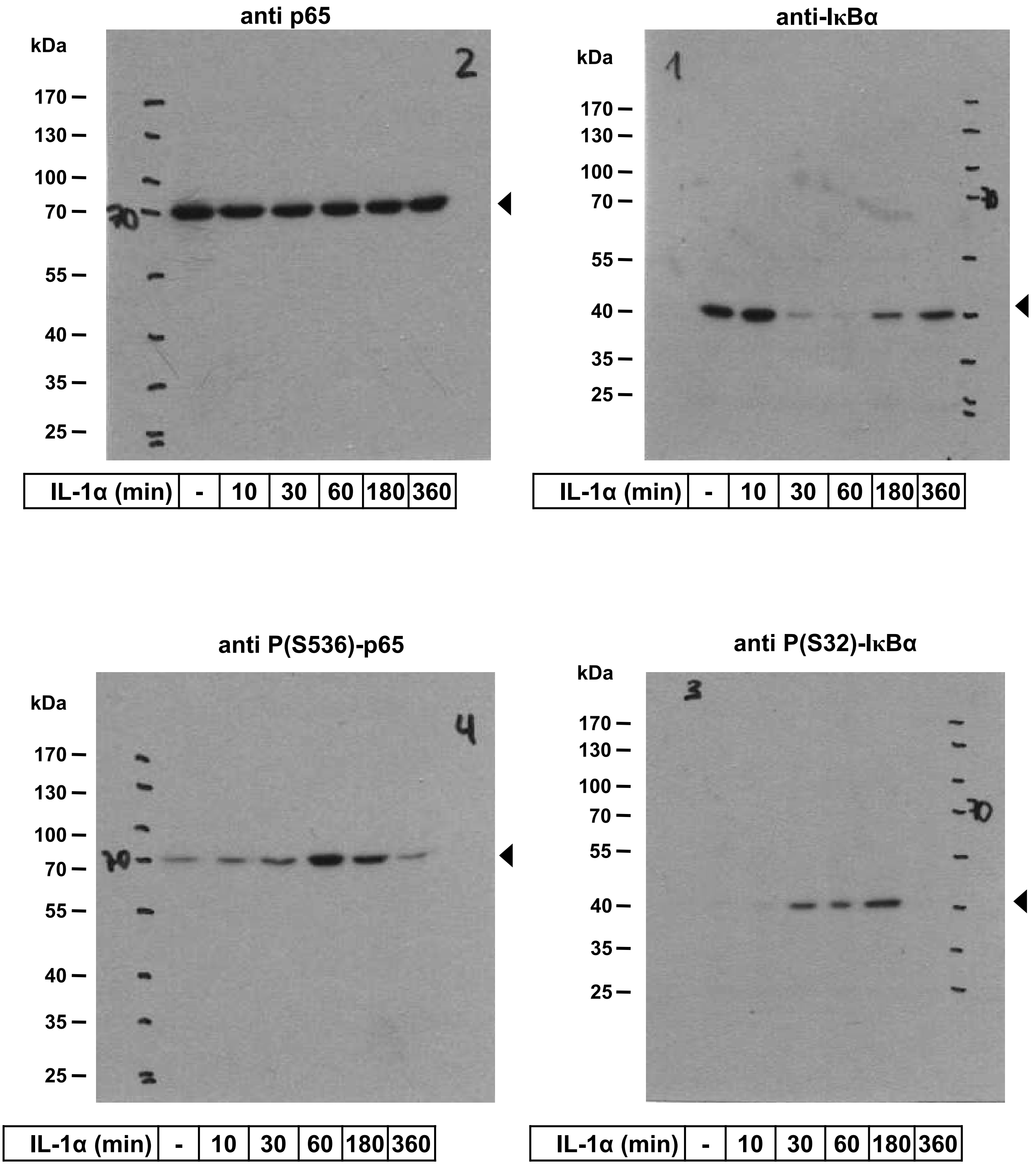
anti p65
kDa
170
130
100
70
55
40
35
25
2
IL-1α (min)
-
10
30
60
180
360
anti-IκBα
kDa
170
130
100
70
55
40
35
25
1
IL-1α (min)
-
10
30
60
180
360
anti P(S536)-p65
kDa
170
130
100
70
55
40
35
25
4
IL-1α (min)
-
10
30
60
180
360
anti P(S32)-IκBα
kDa
170
130
100
70
55
40
35
25
3
IL-1α (min)
-
10
30
60
180
360

Figure 3A

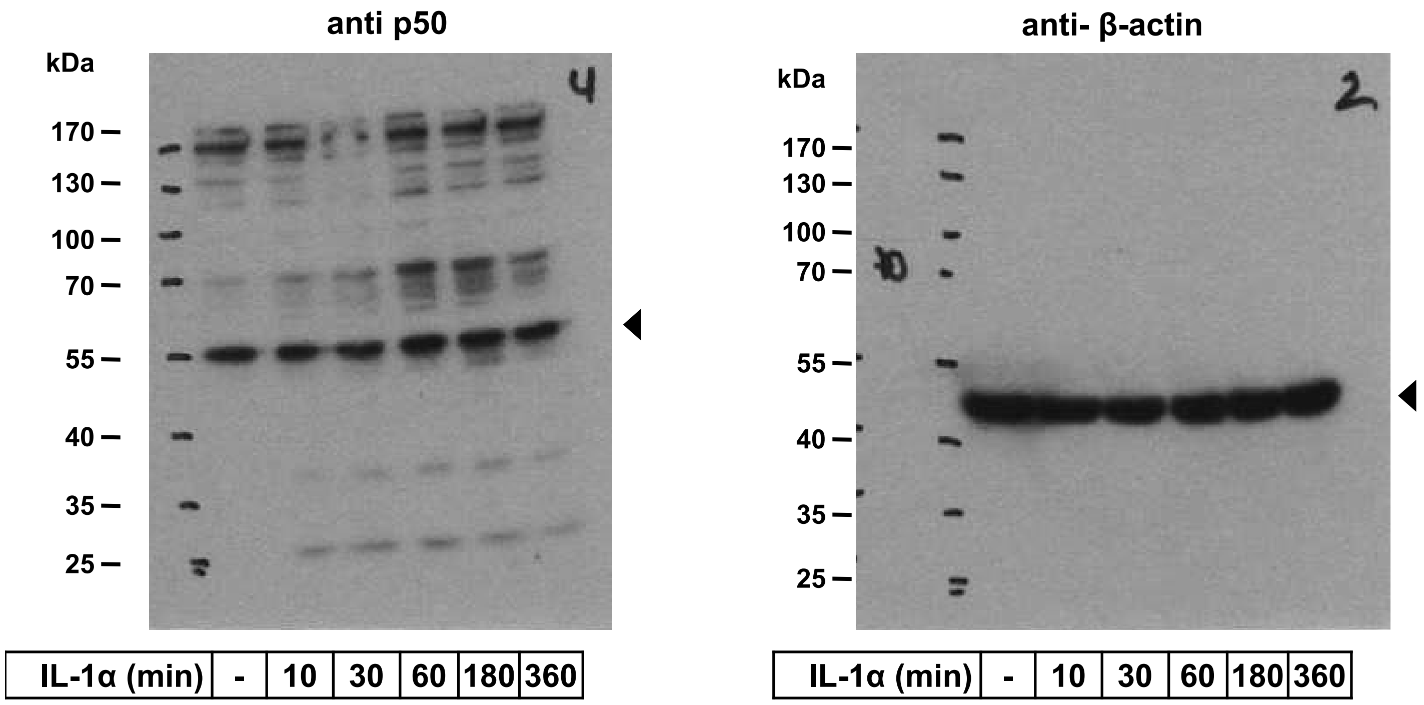
anti p50
kDa
170
130
100
70
55
40
35
25
anti- β-actin
kDa
170
130
100
70
55
40
35
25
IL-1α (min)
-
10
30
60
180
360
IL-1α (min)
-
10
30
60
180
360

Figure 3B

anti p65 (short exposure)

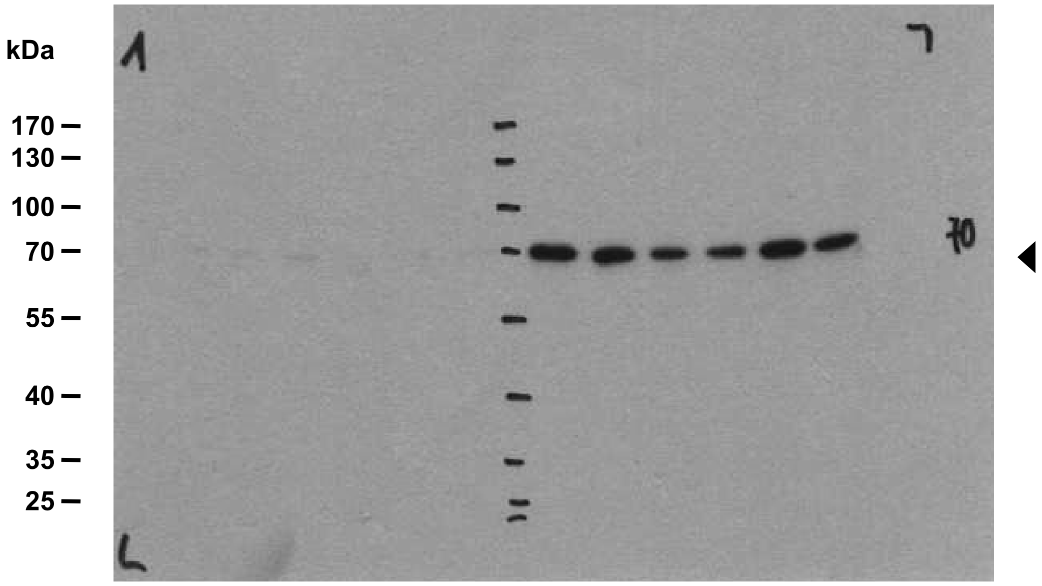

IκBα-IP								+	+	+	+	+	+
IgG-IP	+	+	+	+	+	+							
IL-1α (min)	-	10	30	60	180	360		-	10	30	60	180	360

anti p65 (long exposure)

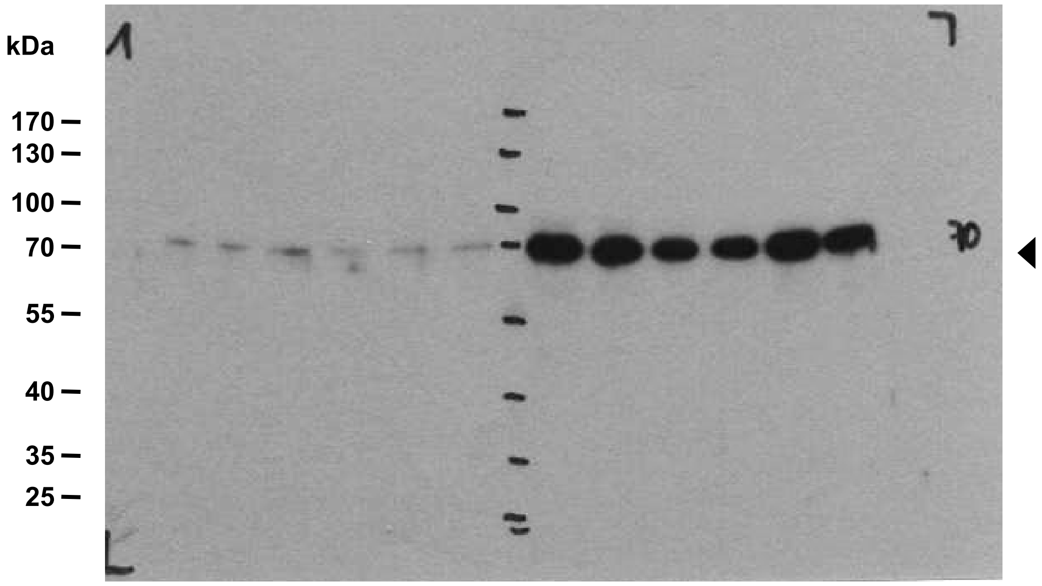

IκBα-IP								+	+	+	+	+	+
IgG-IP	+	+	+	+	+	+							
IL-1α (min)	-	10	30	60	180	360		-	10	30	60	180	360

Figure 3B

anti-IκBα

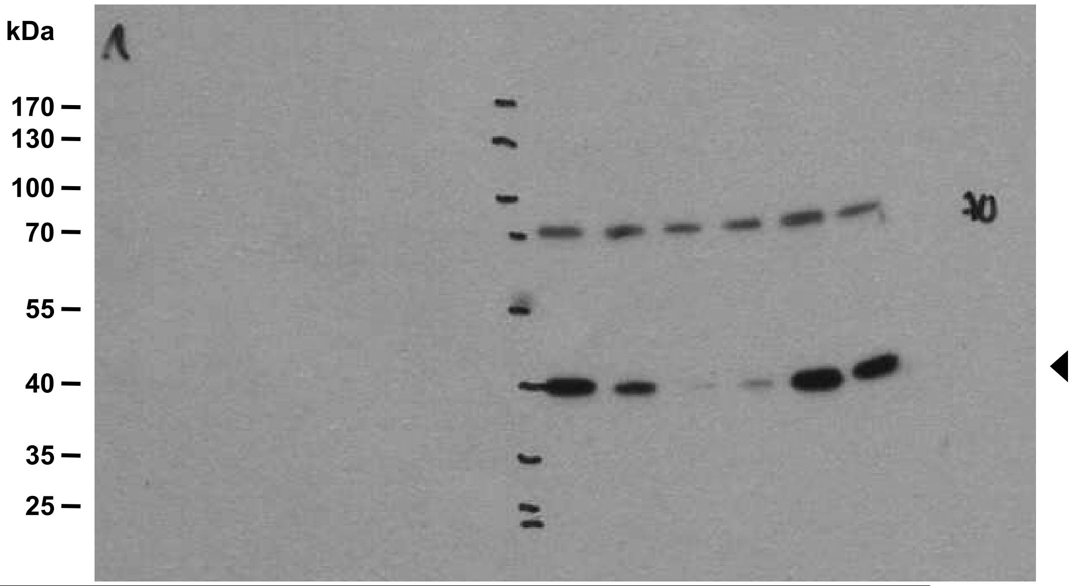

IκBα-IP								+	+	+	+	+	+
IgG-IP	+	+	+	+	+	+							
IL-1α (min)	-	10	30	60	180	360		-	10	30	60	180	360

Figure 4A

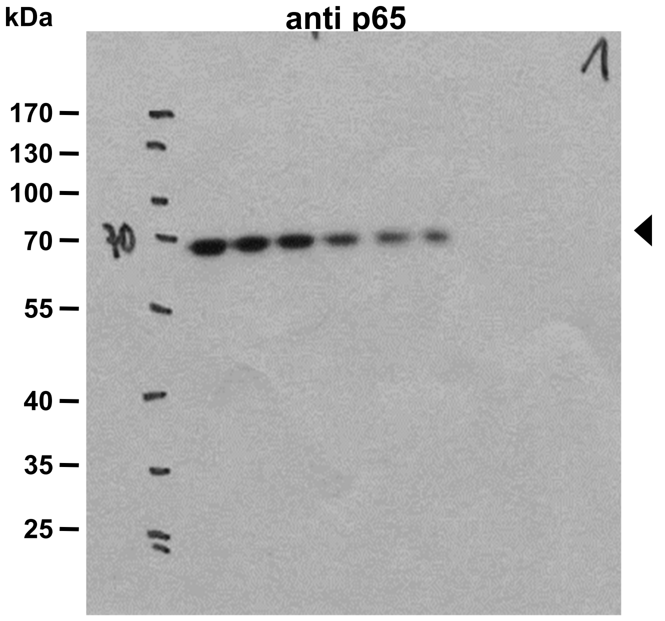

parental	+	+	+						
pSUPER.puro				+	+	+			
shp65							+	+	+
IL-1α (min)		30	60		30	60		30	60

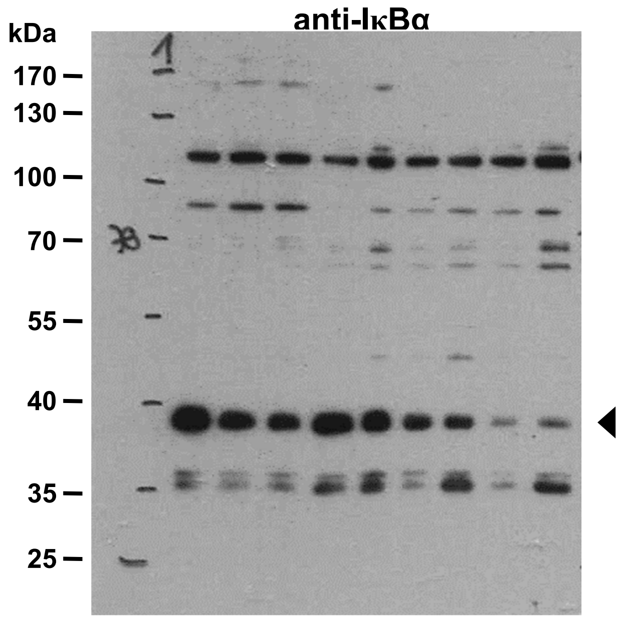

parental	+	+	+						
pSUPER.puro				+	+	+			
shp65							+	+	+
IL-1α (min)		30	60		30	60		30	60

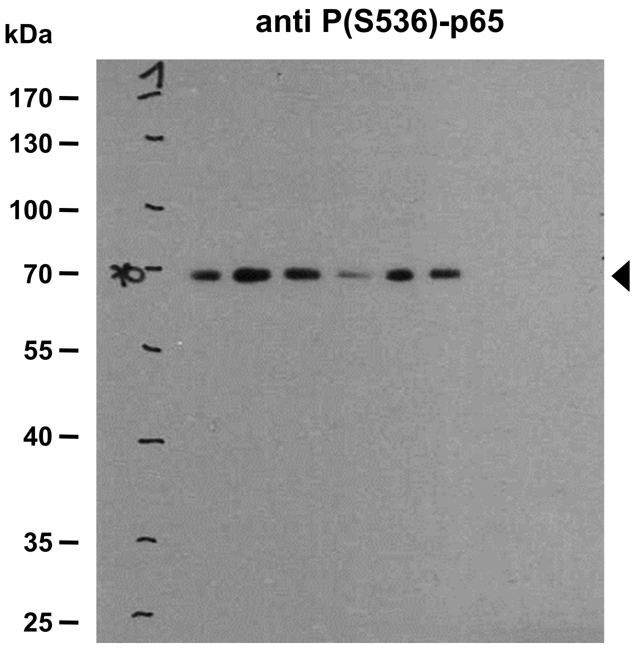

parental	+	+	+						
pSUPER.puro				+	+	+			
shp65							+	+	+
IL-1α (min)		30	60		30	60		30	60

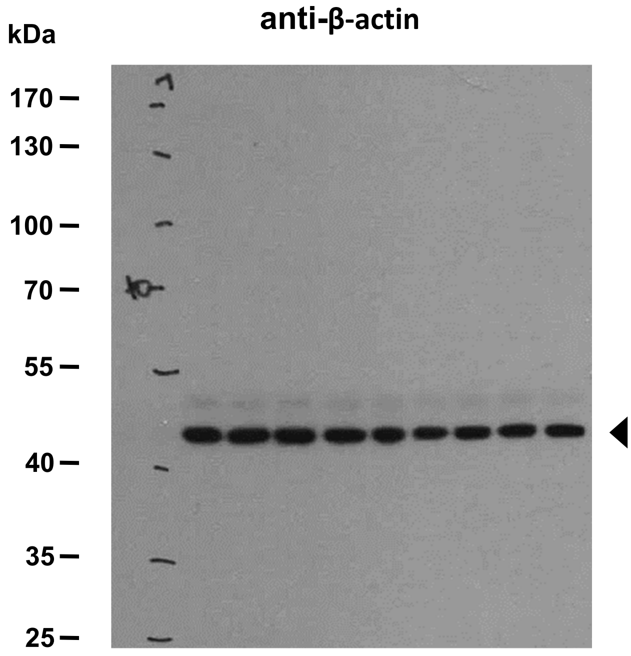

parental	+	+	+						
pSUPER.puro				+	+	+			
shp65							+	+	+
IL-1α (min)		30	60		30	60		30	60

Experiment #1 for figure 7A and densitometry fig. 7B

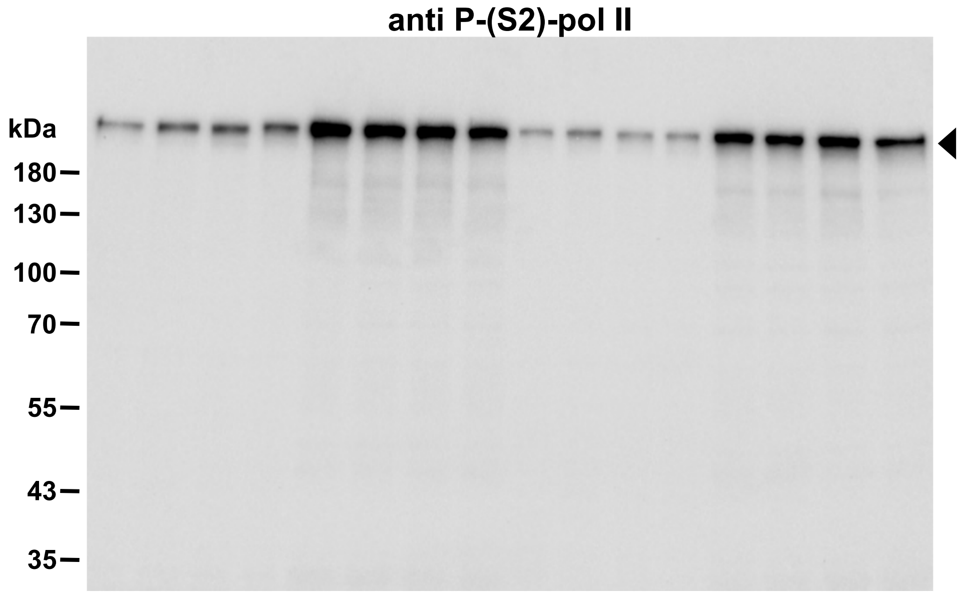

	cytosol				nucleus				cytosol				nucleus			
px459	+	+	+	+	+	+	+	+								
ΔDCP1a									+	+	+	+	+	+	+	+
IL-1 [h]	-	.5	1	3	-	.5	1	3	-	.5	1	3	-	.5	1	3

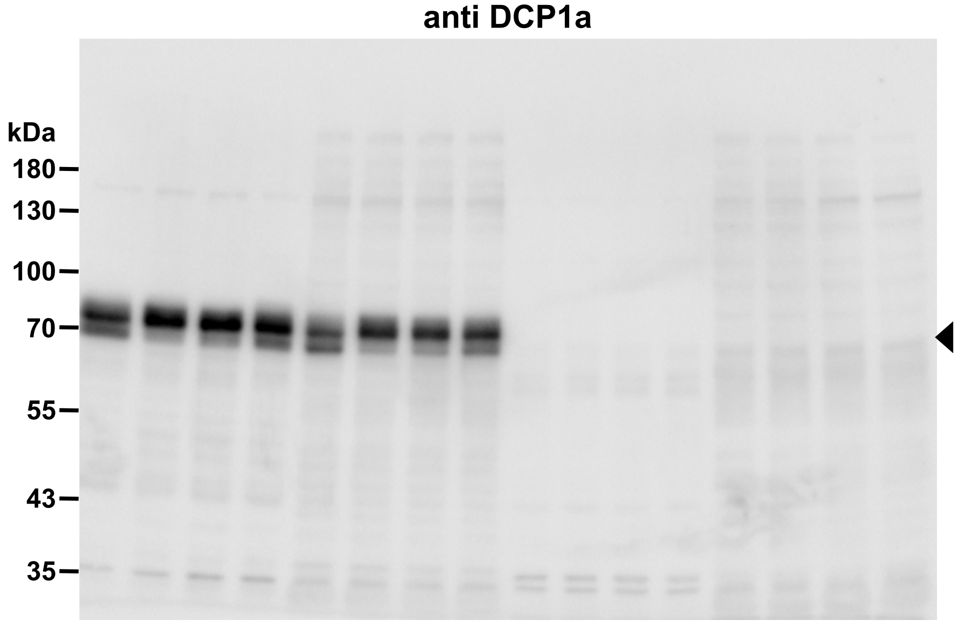

	cytosol				nucleus				cytosol				nucleus			
px459	+	+	+	+	+	+	+	+								
ΔDCP1a									+	+	+	+	+	+	+	+
IL-1 [h]	-	.5	1	3	-	.5	1	3	-	.5	1	3	-	.5	1	3

Experiment #1 for figure 7A and densitometry fig. 7B

anti p65

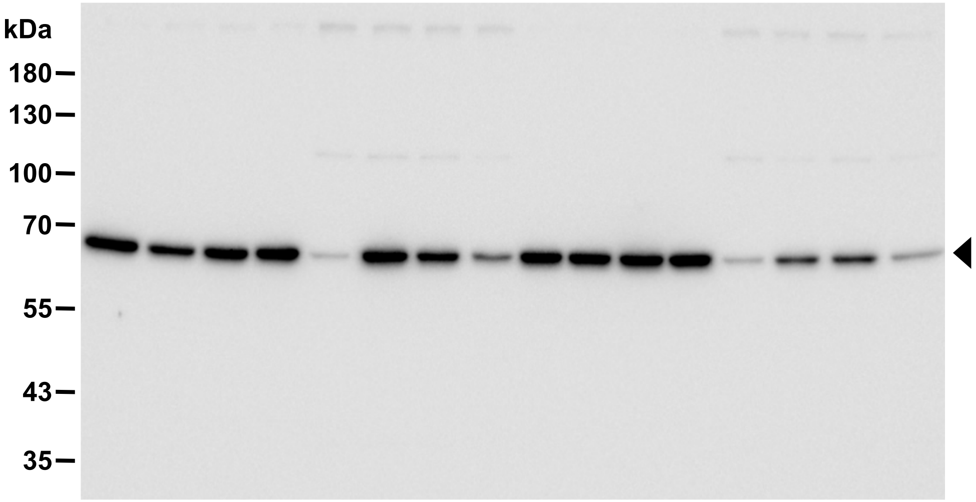

	cytosol				nucleus				cytosol				nucleus			
px459	+	+	+	+	+	+	+	+								
ΔDCP1a									+	+	+	+	+	+	+	+
IL-1 [h]	-	.5	1	3	-	.5	1	3	-	.5	1	3	-	.5	1	3

anti P-(S365)-p65

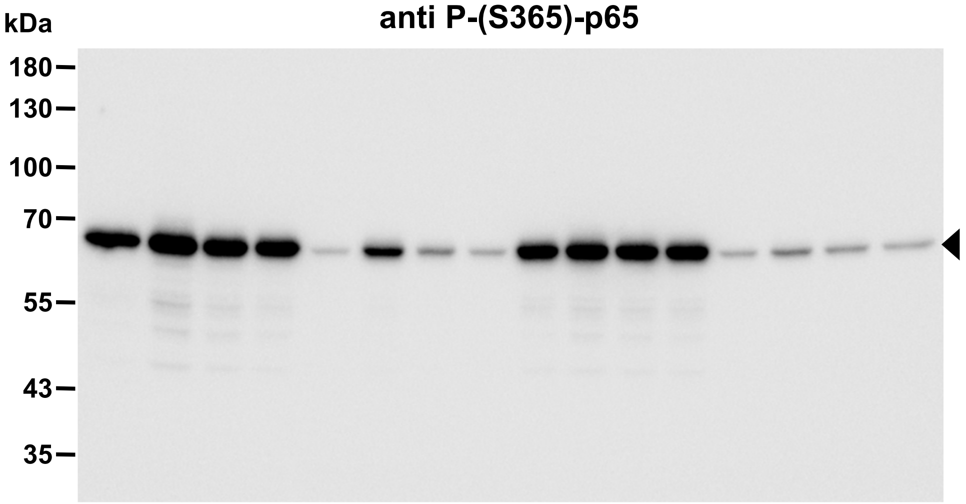

	cytosol				nucleus				cytosol				nucleus			
px459	+	+	+	+	+	+	+	+								
ΔDCP1a									+	+	+	+	+	+	+	+
IL-1 [h]	-	.5	1	3	-	.5	1	3	-	.5	1	3	-	.5	1	3

Experiment #1 for figure 7A and densitometry fig. 7B

anti tubulin

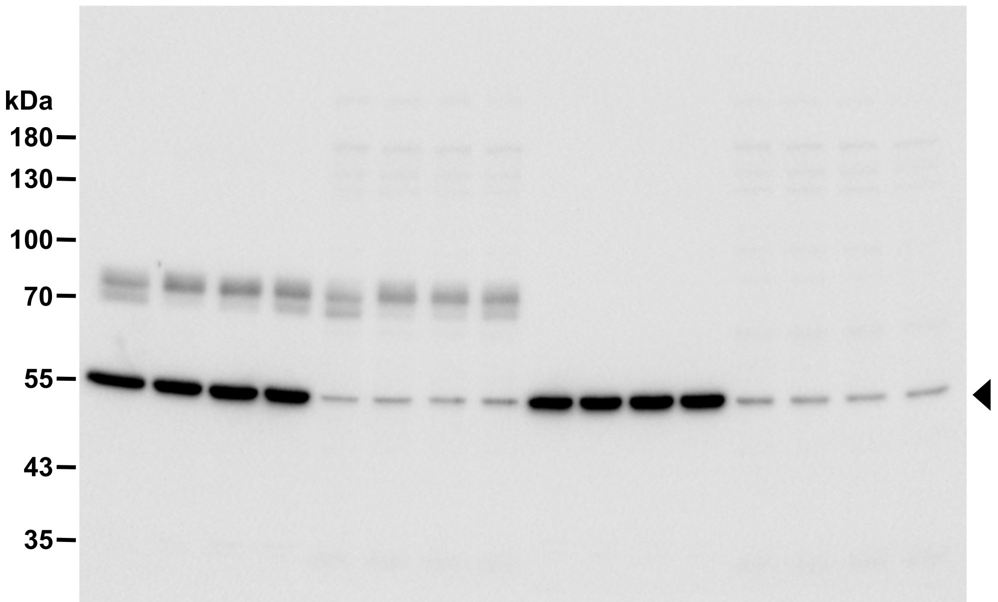

	cytosol				nucleus				cytosol				nucleus			
px459	+	+	+	+	+	+	+	+								
ΔDCP1a									+	+	+	+	+	+	+	+
IL-1 [h]	-	.5	1	3	-	.5	1	3	-	.5	1	3	-	.5	1	3

anti ß-actin

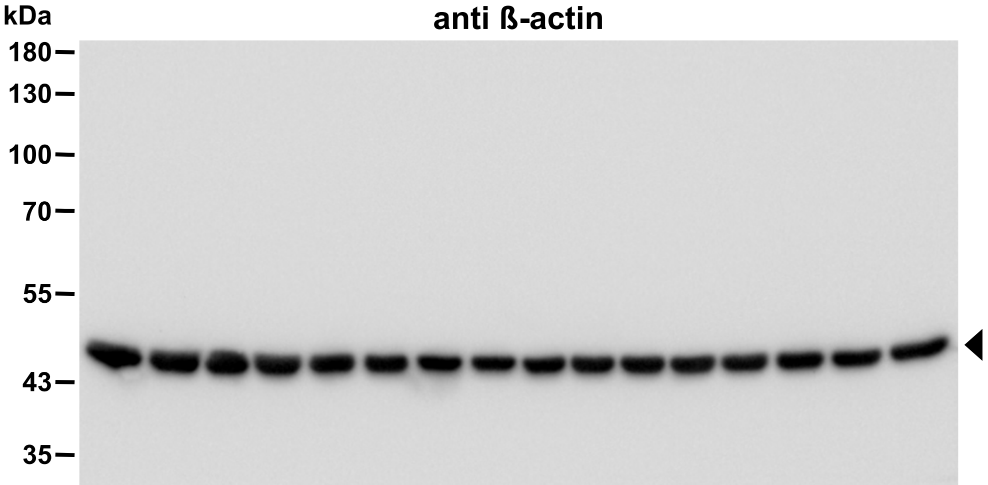

	cytosol				nucleus				cytosol				nucleus			
px459	+	+	+	+	+	+	+	+								
ΔDCP1a									+	+	+	+	+	+	+	+
IL-1 [h]	-	.5	1	3	-	.5	1	3	-	.5	1	3	-	.5	1	3

Experiment #2 for densitometry fig. 7B

anti P-(S2)-pol II

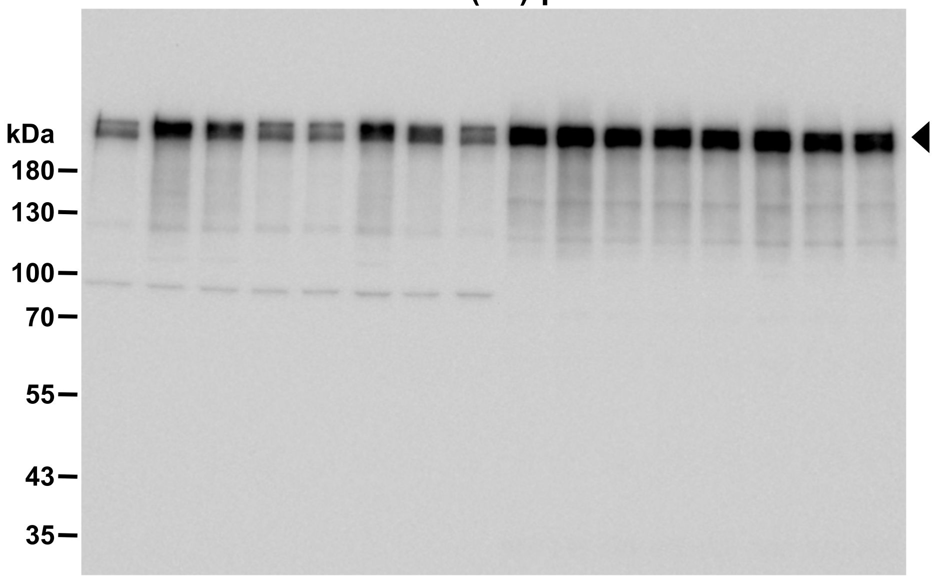

	cytosol								nucleus							
px459	+	+	+	+					+	+	+	+				
ΔDCP1a					+	+	+	+					+	+	+	+
IL-1 [h]	-	.5	1	3	-	.5	1	3	-	.5	1	3	-	.5	1	3

anti DCP1a

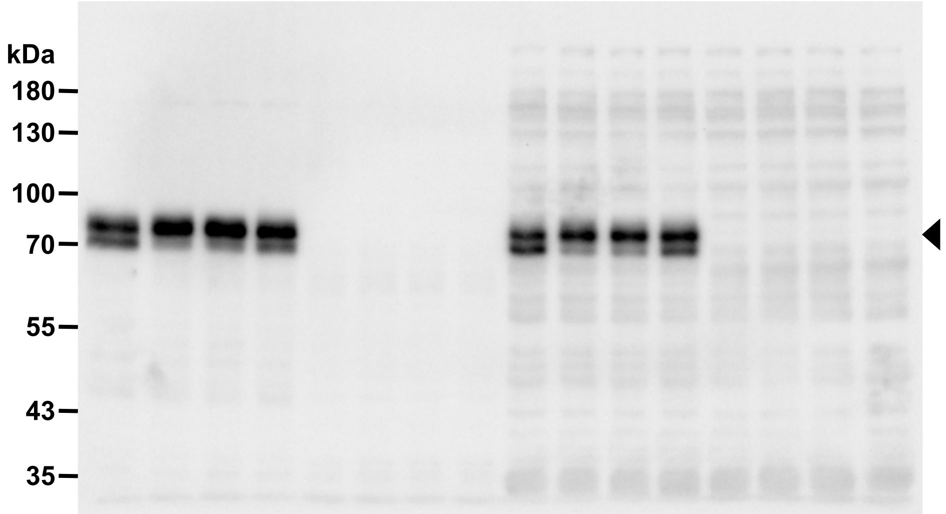

	cytosol								nucleus							
px459	+	+	+	+					+	+	+	+				
ΔDCP1a					+	+	+	+					+	+	+	+
IL-1 [h]	-	.5	1	3	-	.5	1	3	-	.5	1	3	-	.5	1	3

Experiment #2 for densitometry fig. 7B

anti p65

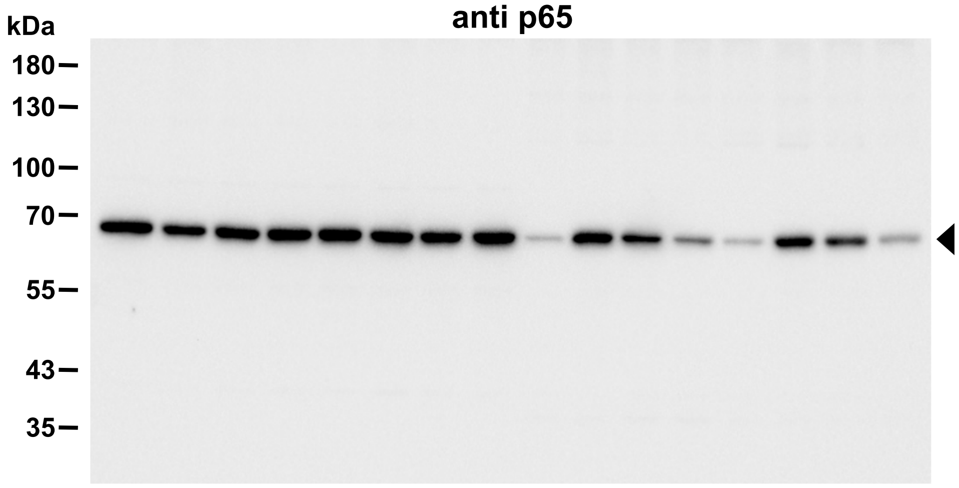

	cytosol								nucleus							
px459	+	+	+	+					+	+	+	+				
ΔDCP1a					+	+	+	+					+	+	+	+
IL-1 [h]	-	.5	1	3	-	.5	1	3	-	.5	1	3	-	.5	1	3

anti P-(S365)-p65

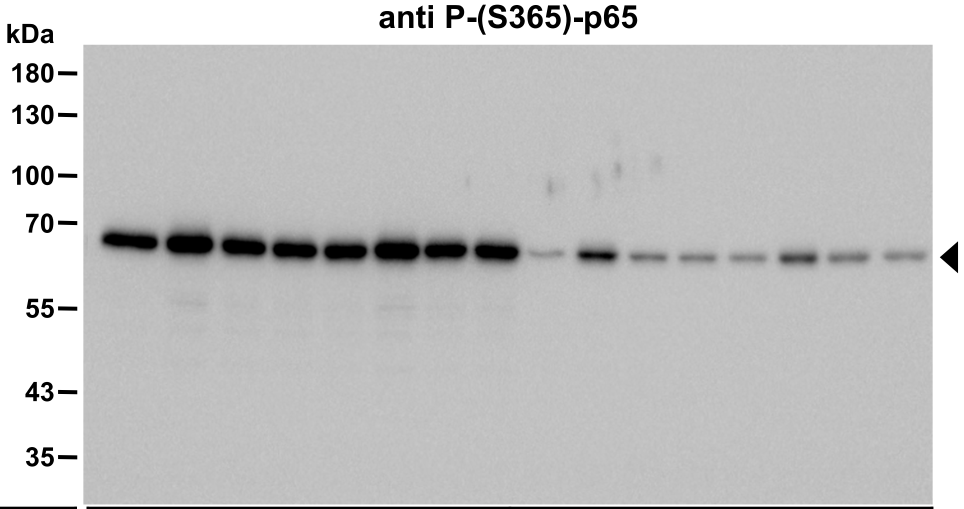

	cytosol								nucleus							
px459	+	+	+	+					+	+	+	+				
ΔDCP1a					+	+	+	+					+	+	+	+
IL-1 [h]	-	.5	1	3	-	.5	1	3	-	.5	1	3	-	.5	1	3

Experiment #2 for densitometry fig. 7B

anti tubulin

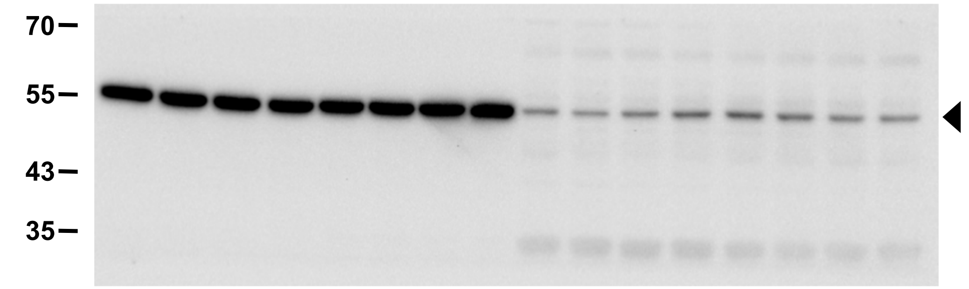

	cytosol								nucleus							
px459	+	+	+	+					+	+	+	+				
ΔDCP1a					+	+	+	+					+	+	+	+
IL-1 [h]	-	.5	1	3	-	.5	1	3	-	.5	1	3	-	.5	1	3

anti ß-actin

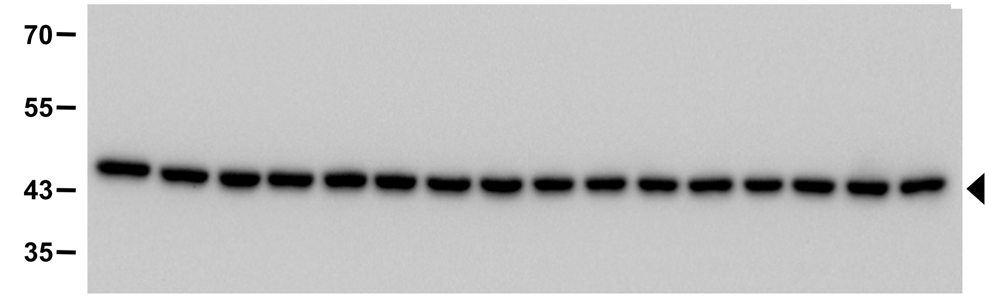

	cytosol								nucleus							
px459	+	+	+	+					+	+	+	+				
ΔDCP1a					+	+	+	+					+	+	+	+
IL-1 [h]	-	.5	1	3	-	.5	1	3	-	.5	1	3	-	.5	1	3

Figure 8H

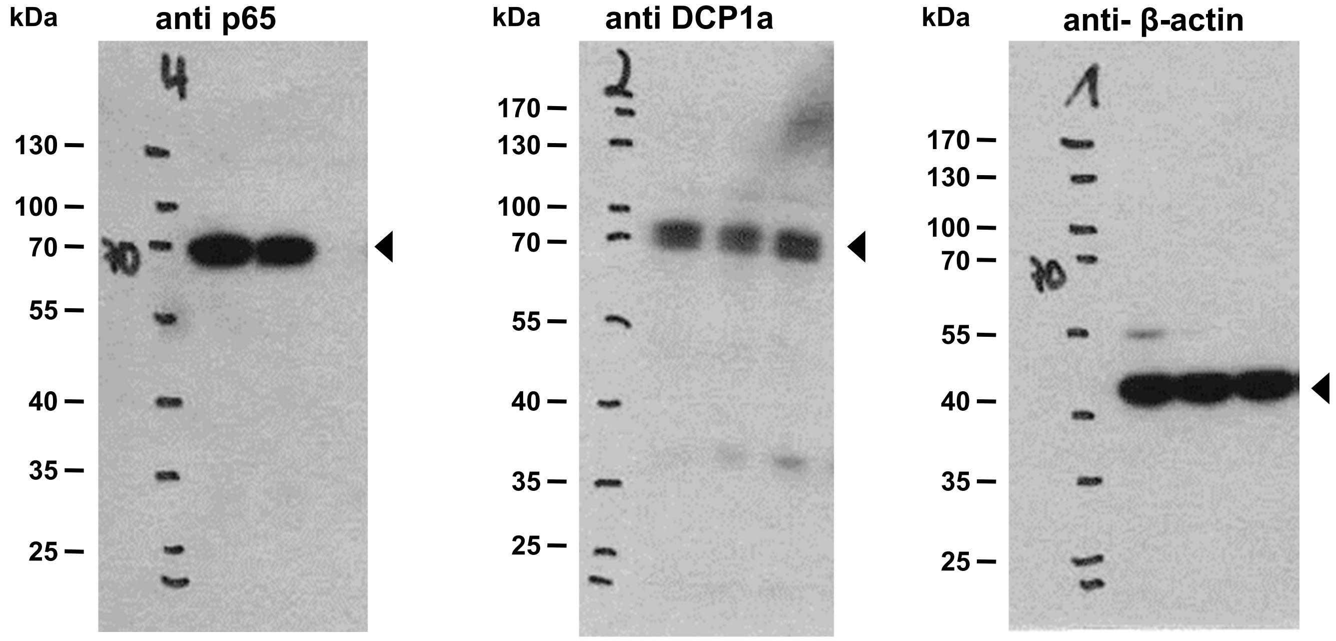

parental	+		
pSUPER.puro		+	
shp65			+

parental	+		
pSUPER.puro		+	
shp65			+

parental	+		
pSUPER.puro		+	
shp65			+
